# Lingguizhugan Decoction Ameliorates MASLD by Modulating the Gut Microbiota and Enriching Non-12-OH Bile Acids to Activate TGR5-Mediated Thermogenesis

**DOI:** 10.3390/ph19040523

**Published:** 2026-03-24

**Authors:** Yun-Hong Sun, Pei-Lun Ding, Xue Wang, Yi-Rong Wang, Ming-Zhe Zhu, Kai Wang, Liang Dai, Yan-Qi Dang, Guang Ji, Meng Li, Wen-Jun Zhou

**Affiliations:** 1 Institute of Digestive Diseases, Shanghai University of Traditional Chinese Medicine, Shanghai 200032, China; syh_010122@163.com (Y.-H.S.); llaipp1667735673@163.com (P.-L.D.); wx130218@163.com (X.W.); wangyirong404@163.com (Y.-R.W.); yajlzs123@163.com (L.D.); dangyanqi9022@126.com (Y.-Q.D.); jg@shutcm.edu.cn (G.J.); 2 School of Public Health, Shanghai University of Traditional Chinese Medicine, Shanghai 201203, China; zhumingzhe@sibs.ac.cn; 3 CAS Engineering Laboratory for Nutrition, Shanghai Institute of Nutrition and Health, University of Chinese Academy of Sciences, Shanghai 200031, China; 4 Experiment Center for Science and Technology, Shanghai University of Traditional Chinese Medicine, Shanghai 201203, China; jeffrey_wangkai@hotmail.com; 5 State Key Laboratory of Integration and Innovation of Classical Formula and Modern Chinese Medicine, Shanghai 201203, China; 6 School of Biological Sciences, Nanyang Technological University, Singapore 637551, Singapore

**Keywords:** Lingguizhugan decoction, metabolic dysfunction–associated steatotic liver disease, gut–liver axis, non-12-OH BAs, gut microbiota, TGR5, thermogenesis

## Abstract

**Objective**: Based on previous findings on the Lingguizhugan (LGZG)-mediated gut–liver axis, this study clarifies the therapeutic mechanisms of LGZG in metabolic dysfunction-associated steatotic liver disease (MASLD), with a focus on the gut microbiota–bile acid–TGR5 (GPBAR1) axis. **Methods**: C57BL/6J mice were fed a high-fat diet (HFD) for 8 weeks to induce MASLD, followed by 4-week LGZG intervention (21.57 g/kg/day, oral gavage). Metabolic phenotypes, gut microbiota (16S rRNA sequencing), serum/hepatic bile acids (targeted metabolomics), and molecular targets (qPCR/Western blot) were analyzed. **Results**: LGZG significantly alleviated HFD-induced obesity, insulin resistance, and hepatic steatosis, while enhancing whole-body energy expenditure (increased oxygen consumption (VO_2_), and heat production (*p* < 0.05). It also reduced serum ALT (*p* < 0.001) and AST levels (*p* < 0.01). Mechanistically, LGZG remodeled the gut microbiota, specifically increasing *Akkermansia*, *Bifidobacterium* and *Lachnospiraceae*_NK4A236_group while decreasing *Lactobacillus*. This shift inhibited the intestinal FXR-Fgf15 axis, concurrently activating the hepatic alternative bile acid synthesis pathway (upregulating CYP27A1 and CYP7B1 protein expression; *p* < 0.001 and *p* < 0.01, respectively). Consequently, systemic accumulation of non-12α-hydroxylated bile acids (non-12-OH BAs) such as hyocholic acid (HCA) and 7-ketolithocholic acid (7-ketoLCA) occurred—known TGR5 agonists and intestinal FXR antagonists. These changes elevated serum GLP-1 levels (*p* < 0.05) and activated adipose TGR5-cAMP/PKA/CREB signaling. The metabolic benefits primarily originated from non-12-OH BAs enrichment and TGR5-mediated adipose browning, not hepatic FXR activation. **Conclusions**: Our findings show that LGZG ameliorates MASLD by remodeling bile acid profiles via intestinal FXR-Fgf15 axis inhibition and hepatic alternative synthesis pathway activation. This study highlights the TGR5-targeting properties of LGZG, providing a mechanistic basis for its therapeutic use in metabolic disorders.

## 1. Introduction

MASLD, the recently redefined entity replacing nonalcoholic fatty liver disease (NAFLD), currently affects approximately 25–30% of the adult population globally, making it the most prevalent chronic liver disorder worldwide [1,2,3]. As a hepatic manifestation of metabolic syndrome, MASLD is strongly associated with obesity, type 2 diabetes mellitus, and often progresses from simple steatosis to metabolic dysfunction–associated steatohepatitis (MASH), cirrhosis, and hepatocellular carcinoma [1]. Despite the landmark approval of resmetirom—a thyroid hormone receptor-β (TRβ) agonist—as the first disease-modifying therapy [4], monotherapy remains inadequate for MASLD’s heterogeneity. Other investigational agents like FXR agonists face limitations due to adverse effects such as severe pruritus and dyslipidemia [5], underscoring the urgent need for safer, multi-target strategies.

Emerging evidence suggests that gut microbiota dysbiosis serves as a crucial environmental trigger for the pathogenesis of MASLD [6]. Significantly, recent investigations in human cohorts and gnotobiotic mice have causally associated the depletion of *Akkermansia muciniphila* and *Lactobacillus* spp. with compromised gut barrier integrity, endotoxemia, and hepatic inflammation, which are pivotal factors driving the progression of MASLD [7]. Beyond these localized gut-liver effects, contemporary perspectives emphasize that gut microbiota actively participate in systemic metabolic regulation through organ-level nutrient sensing mechanisms. In this framework, specialized enteroendocrine cells (EECs) detect luminal microbial signals and translate them into hormonal outputs (e.g., GLP-1, PYY, CCK), coordinating feeding behavior and energy homeostasis across the brain, liver, and adipose tissue [8].

Within this integrated host-microbe metabolic network, bile acids (BAs) emerge as critical endogenous mediators linking microbial activity to systemic energy sensing. Formerly regarded as mere lipid emulsifiers, BAs are currently recognized as potent endocrine signaling molecules that regulate systemic metabolic homeostasis, encompassing glucose and lipid metabolism [9]. This systemic regulation is increasingly perceived as an integrated host-microbe interaction, in which microbial metabolites, including secondary bile acids, coordinate energy expenditure, thermogenesis, and insulin sensitivity across multiple organs [8].

Gut microbial enzymes, particularly bile salt hydrolases (BSH) and 7α-dehydroxylases, transform primary bile acids into secondary forms, significantly enriching the diversity of the bile acid pool [10]. Notably, microbial 7α-dehydroxylation predominantly generates secondary non-12-OH BAs, such as lithocholic acid (LCA), which confer enhanced metabolic advantages through selective receptor activation [11].

Bile acid biosynthesis occurs via two major pathways: (1) the classical (neutral) pathway (CYP7A1/CYP8B1-dependent), producing 12α-hydroxylated bile acids (12-OH BAs) like cholic acid (CA); and (2) the alternative (acidic) pathway (CYP27A1/CYP7B1-driven), generating non-12-OH BAs including chenodeoxycholic acid (CDCA) [9,12]. Accumulating evidence demonstrates that non-12-OH BAs and their metabolites exert potent metabolic benefits, particularly through activation of key receptors, including the FXR and TGR5 [11,13]. Structure-activity analyses reveal TGR5 exhibits preferential responsiveness to hydrophobic BAs, with secondary non-12-OH BAs (e.g., LCA, a CDCA metabolite) displaying especially potent agonist activity [12,14].

This ligand-receptor interaction underpins TGR5’s regulation of systemic energy homeostasis [15]. TGR5 activation in adipose tissues triggers the cAMP–PKA–CREB signaling cascade [16], upregulating uncoupling protein 1 (UCP1) and mitochondrial biogenesis regulator PGC-1α. This promotes adipose thermogenesis and protects against diet-induced metabolic dysfunction [17]. Consequently, selective enrichment of non-12-OH BAs, particularly potent TGR5 agonists including LCA and related secondary bile acids derived from the alternative pathway, represents a promising strategy. Despite these potential, targeted modulation of the “gut microbiota–alternative bile acid pathway–non-12-OH BA” axis for adipose TGR5 remains underexplored.

Traditional Chinese medicine (TCM) offers multi-target therapeutic potential for MASLD [16]. LGZG—a classical formula from “*Jin Gui Yao Lue*”—comprises *Poria cocos* (Schw.) Wolf (Fuling), *Cinnamomum cassia* Presl (Guizhi), *Atractylodes macrocephala* Koidz. (Baizhu), and *Glycyrrhiza uralensis* Fisch. (Gancao). Our preliminary research has confirmed that LGZG has various different effects against MASLD. At the hepatic level, LGZG counteracts steatosis by inhibiting lipogenesis (regulated by PPP1R3C/SREBP-1c/ACC/HMGCR) and the enhancement of β-oxidation (mediated by TRβ1/CPT1A) [18,19]. Epigenetically, it modulates DNA/RNA methylation (involving ALKBH1-PSD4; m6A-SOCS2-JAK/STAT) to restore lipid transport and insulin sensitivity [20,21]. Critically, LGZG reduces *Firmicutes/Bacteroidetes* ratio, enriches *Akkermansia*, and reinforces gut barrier [20,21,22]. Notably, given the established role of LGZG in remodeling gut microbiota and the emerging paradigm of microbiota-driven systemic nutrient sensing, LGZG’s potential impact on adipose thermogenesis via the gut microbiota–non-12-OH BA–TGR5 axis warrants systematic investigation.

Here, we hypothesize that LGZG alleviates MASLD by remodeling gut microbiota to activate the alternative BA pathway, thereby enriching TGR5-activating non-12-OH BAs (e.g., LCA) and stimulating adipose thermogenesis. Using a high-fat diet-induced MASLD model, we investigate this novel “gut microbiota–alternative BA pathway–non-12-OH BAs–adipose TGR5 thermogenesis” axis within the broader context of systemic nutrient sensing and interorgan metabolic coordination.

## 2. Results

### 2.1. Effects of LGZG on Body Weight, Serum Liver Injury Markers, and Glucose Homeostasis in HFD-Induced MASLD Mice

**Figure 1:**
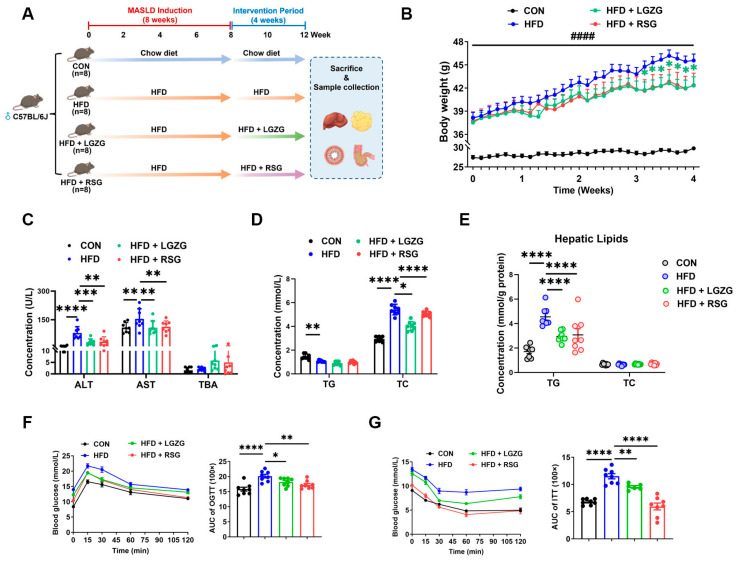
LGZG ameliorates metabolic dysfunction in HFD-induced murine MASLD. (**A**) Schematic representation of the experimental study design (*n* = 8 mice per group). C57BL/6J mice were fed chow (CON) or HFD for 8 weeks to induce MASLD, followed by a 4-week therapeutic intervention with vehicle, LGZG (21.57 g/kg/day), or RSG (10 mg/kg/day) while continuing HFD. (**B**) Body weight changes during the intervention period. (**C**) Serum AST, ALT and TBA levels. (**D**) Serum TG and TC levels. (**E**) Hepatic TG and TC contents. (**F**,**G**) OGTT and ITT curves with AUC quantification. Data are presented as mean ± SEM (*n* = 8 mice/group). Statistical significance was determined by two-way ANOVA with Dunnett’s test (**B**), one-way ANOVA with Dunnett’s test (**C**–**E**), or two-way ANOVA with repeated measures and Dunnett’s test (**F**,**G**). * *p* < 0.05, ** *p* < 0.01, *** *p* < 0.001, **** *p* < 0.0001 vs. HFD group; ^####^ *p* < 0.0001 vs. CON.

To explore the therapeutic efficacy of LGZG in MASLD, C57BL/6J mice were fed a HFD for 8 weeks to induce MASLD, followed by a 4-week intervention phase period with LGZG or RSG (Figure 1A; *n* = 8 mice per group). As shown in Figure 1B, 8 weeks of HFD feeding induced a sustained and progressive increase in body weight compared with chow-fed controls (*p* < 0.0001). From the fourth week of the intervention period, LGZG treatment significantly suppressed further body weight gain (*p* < 0.05 vs. HFD). Serum liver injury marker assays revealed that HFD feeding significantly elevated serum ALT and AST levels (*p* < 0.001 and *p* < 0.01). LGZG intervention significantly reduced both serum ALT and AST (*p* < 0.001 and *p* < 0.05, vs. HFD) (Figure 1C).

Assessment of systemic lipid metabolism revealed that HFD feeding significantly reduced serum TG levels (*p* < 0.05 vs. CON), a phenomenon previously reported in advanced MASLD models suggesting impaired hepatic VLDL-TG assembly and export [23]. In contrast, serum TC levels were markedly elevated (*p* < 0.0001). LGZG intervention significantly reduced serum TC (*p* < 0.05 vs. HFD), whereas RSG showed comparable efficacy (*p* < 0.0001) (Figure 1D). At the hepatic level, HFD feeding triggered profound lipid accumulation, as evidenced by a pronounced increase in hepatic TG content (*p* < 0.0001 vs. CON). Both LGZG and RSG treatments markedly attenuated hepatic TG accumulation (*p* < 0.0001 vs. HFD) (Figure 1E).

Systemic glucose homeostasis and insulin sensitivity were next evaluated using oral glucose tolerance test (OGTT) and insulin tolerance test (ITT). HFD feeding markedly impaired glucose tolerance, as evidenced by elevated glycemic excursions and increased AUC values (*p* < 0.0001 vs. CON). LGZG administration significantly improved glucose tolerance (*p* < 0.05 vs. HFD), while RSG intervention showed a comparable ameliorative effect (*p* < 0.01 vs. HFD) (Figure 1F). In the ITT, HFD feeding significantly elevated blood glucose levels (*p* < 0.0001 vs. CON). Both LGZG and RSG treatments markedly reduced glucose levels compared to the HFD group (*p* < 0.01 and *p* < 0.0001, respectively) (Figure 1G).

### 2.2. LGZG Attenuates Hepatic Steatosis and Pathological Damage

**Figure 2:**
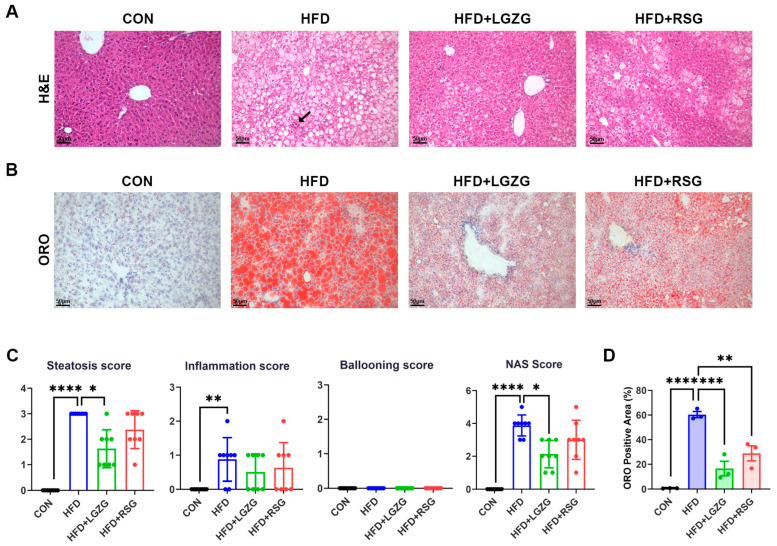
LGZG attenuates hepatic steatosis and improves hepatic histopathology in HFD-induced MASLD mice. (**A**) Representative hematoxylin and eosin (H&E)-stained liver sections (scale bar = 50 μm, magnification: 200×). Black arrows indicate scattered inflammatory foci in the HFD group. (**B**) Representative Oil Red O-stained liver sections (scale bar = 50 μm, magnification: 200×). (**C**) Histopathological scoring using the non-alcoholic fatty liver disease (NAFLD) Activity Score (NAS) system, comprising steatosis, lobular inflammation, and hepatocyte ballooning components. (**D**) Quantification of hepatic lipid accumulation, presented as Oil Red O-positive area (%) (*n* = 3 fields per mouse). Data are presented as mean ± SEM (*n* = 8 mice/group, with 3 fields analyzed per mouse for Oil Red O staining). Statistical significance was determined by the Kruskal–Wallis test with Dunn’s multiple comparisons test (**C**) or one-way ANOVA with Dunnett’s test (**D**). * *p* < 0.05, ** *p* < 0.01, *** *p* < 0.001, **** *p* < 0.0001 vs. HFD group.

Given that LGZG improved hepatic TG accumulation and serum liver injury markers (Section 2.1), we next examined its hepatoprotective effects on HFD-induced hepatic steatosis via histopathological analyses. H&E staining showed that HFD feeding caused marked hepatic steatosis accompanied by inflammatory changes, characterized by macrovesicular lipid droplets (Figure 2A). Consistently, LGZG treatment markedly decreased the total non-alcoholic fatty liver disease (NAFLD) activity score (NAS) relative to HFD controls (NAS reduced from 3.875 ± 0.64 to 2.125 ± 0.83, *p* < 0.05). Notably, the reduction in total NAS was primarily driven by the significant improvement in the hepatic steatosis score rather than the inflammation score, as the latter did not show a statistically significant difference between HFD and LGZG groups (Figure 2C).

Consistent with these findings, Oil Red O staining demonstrated a marked decrease in hepatic lipid accumulation in both LGZG- and RSG-treated mice (Figure 2B). Quantitative analysis further confirmed a 72.67% reduction in the Oil Red O-positive area compared with the HFD group (*p* < 0.001 vs. HFD), with RSG also showing significant efficacy (*p* < 0.01) (Figure 2D).

Collectively, the results in Section 2.1 and Section 2.2 demonstrate that LGZG effectively attenuates HFD-induced MASLD in mice, accompanied by improved systemic glucose homeostasis and insulin sensitivity, as well as reduced hepatic TG accumulation, steatosis, and associated liver pathological damage.

### 2.3. LGZG Reshapes Gut Microbiota and Regulates Bile Acid-Correlated Microbial Function

**Figure 3:**
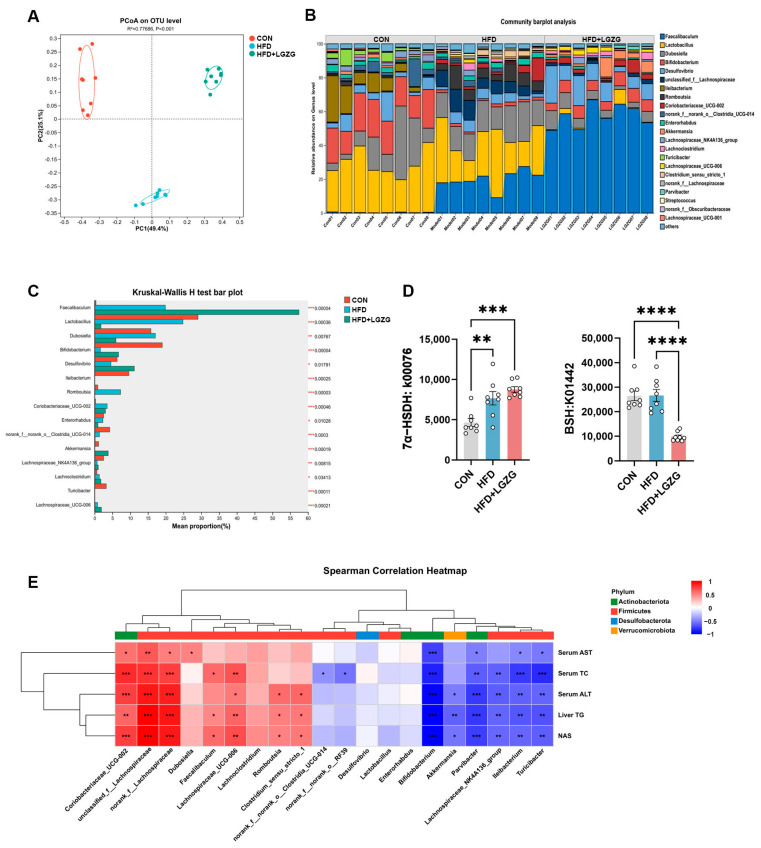
LGZG modifies the composition of gut microbiota and the bile acid–associated functional potential in HFD-induced MASLD mice. (**A**) Beta diversity was analyzed via PCoA based on unweighted UniFrac distances. Beta-diversity differences were evaluated by permutational multivariate analysis of variance (PERMANOVA) for beta diversity and the Kruskal–Wallis H test for intergroup differential taxa. (**B**) A genus-level community bar plot and a relative abundance heatmap were employed to illustrate the distribution of gut microbiota across different groups. (**C**) A bar plot based on the Kruskal–Wallis H test was used to identify intergroup differential microbial taxa at the genus level (only the top 15 most abundant taxa are presented). (**D**) PICRUSt2 functional prediction analysis indicated that LGZG intervention significantly upregulated the functional abundance of microbial enzymes associated with 7α-dehydroxylation (a crucial step for non-12-hydroxylated bile acid synthesis) and downregulated bile salt hydrolase (BSH, the core enzyme mediating conjugated bile acid deconjugation). (**E**) Spearman’s rank correlation heatmap was used to show the relationships between the relative abundance of the top 20 gut microbiota and clinical parameters (serum ALT, AST, and TC; hepatic TG; NAS score). Hierarchical clustering was carried out using the average linkage method. *p* < 0.05 after Benjamini–Hochberg false discovery rate (FDR) correction. Statistical significance was determined by one-way analysis of variance (ANOVA) with Dunnett’s test (**D**). * *p* < 0.05, ** *p* < 0.01, *** *p* < 0.001, **** *p* < 0.0001 vs. HFD group.

Considering that gut microbiota dysbiosis is closely correlated with MASLD progression and BA metabolism, we further explored the impact of LGZG on gut microbiota using 16S rRNA gene sequencing of ileocecal contents. The principal coordinate analysis (PCoA) based on unweighted UniFrac distances demonstrated distinct clustering among the CON, HFD, and HFD + LGZG groups (permutational multivariate analysis of variance [PERMANOVA], *p* < 0.05). Notably, LGZG-treated samples were positioned distinctly from both the CON and HFD groups (Figure 3A), indicating that LGZG induces a unique microbial restructuring rather than a simple reversion to the control state.

At the genus level, community barplot and relative abundance heatmap revealed distinct gut microbiota compositions across the three groups (Figure 3B). Differential abundance analysis (Kruskal–Wallis test) further identified specific taxa altered by LGZG treatment. In particular, *Faecalibacterium*, *Desulfovibrio*, *Akkermansia*, *Bifidobacterium*, and *Lachnospiraceae*_NK4A136 group were significantly increased, while *Lactobacillus*, *Dubosiella*, and *Romboutsia* were markedly decreased relative to HFD controls (Figure 3C).

To further investigate the relationship between these microbial changes and the observed clinical improvements, Spearman’s rank correlation analysis with hierarchical clustering was performed (Figure 3E). Specifically, *Akkermansia* showed significant negative correlations with NAS, hepatic TG, and serum ALT (*p* < 0.05), consistent with its established protective role in metabolic diseases. Notably, *Bifidobacterium* demonstrated strong negative correlations with all clinical parameters (*p* < 0.001), highlighting its robust association with improved metabolic outcomes. However, *Faecalibacterium* exhibited significant positive correlations with NAS, hepatic TG, and serum TC (*p* < 0.05), despite being elevated in both HFD and LGZG groups. This apparent discrepancy suggests that *Faecalibacterium* abundance may primarily reflect luminal nutrient availability or dietary substrate changes—common to both high-fat feeding and LGZG intervention—rather than serving as a direct biomarker of metabolic health. In contrast, *Desulfovibrio* and *Lactobacillus* showed no significant correlations with any metabolic or hepatic injury markers, indicating that their alterations may reflect indirect effects of LGZG treatment rather than direct mechanistic contributions. These correlation patterns support a mechanistic link between LGZG-mediated gut microbiota remodeling and its metabolic and hepatoprotective effects (Figure 3E).

PICRUSt2 functional prediction analysis was performed to assess microbial enzymes involved in BA metabolism. LGZG intervention markedly upregulated the functional abundance of microbial enzymes linked to 7α-dehydroxylation (a key step for non-12-OH BA synthesis), while significantly downregulating bile salt hydrolase (BSH), the core enzyme mediating conjugated BA deconjugation (Figure 3D). These functional shifts suggest that LGZG may promote the production of non-12-OH BAs while potentially enhancing hepatic accumulation of conjugated total BAs.

Collectively, these findings confirm that LGZG elicits profound structural and functional remodeling of the gut microbiota, with selective modulation of BA-metabolizing enzymes. This microbiota-BA axis likely represents a key mechanistic underpinning of LGZG’s therapeutic efficacy against HFD-induced MASLD. Accordingly, we next performed targeted BA metabolomic analyses to investigate whether LGZG-induced microbial remodeling drives alterations in hepatic and systemic BA profiles.

### 2.4. LGZG Promotes Non-12-OH BA Accumulation via Selective Activation of the Alternative Hepatic Bile Acid Synthesis Pathway

**Figure 4:**
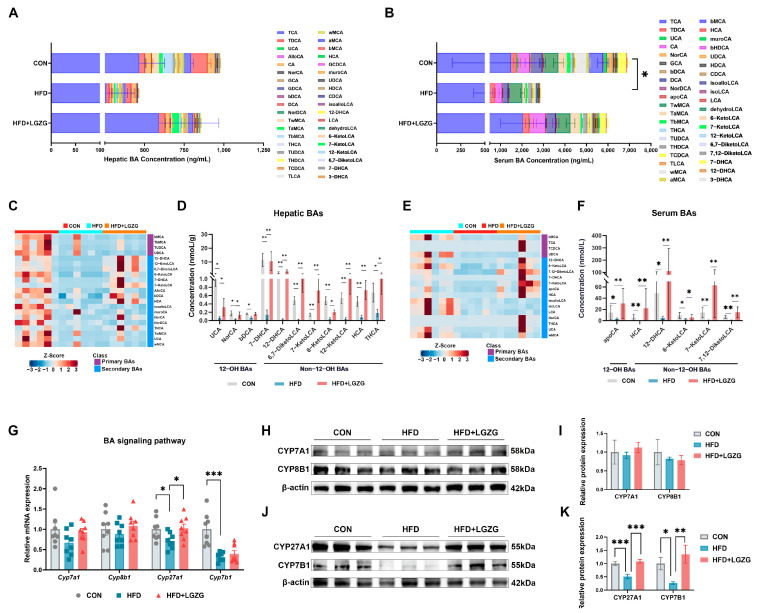
LGZG regulates BA profile and hepatic BA synthesis pathways in HFD-induced MASLD mice. (**A**) Stacked composition of hepatic bile acids (BAs) in CON, HFD, and HFD + LGZG groups. (**B**) Stacked composition of serum BAs in CON, HFD, and HFD + LGZG groups. (**C**) Z-score heatmap of hepatic BA species (primary vs. secondary BAs as indicated). Heatmaps represent Z-score normalized intensities; differential species were screened by VIP > 1 and *p* < 0.05. (**D**) Absolute content of overlapping differential BAs in the liver (intersection of differential BAs from LGZG vs. HFD and HFD vs. CON, mostly non-12α-hydroxylated secondary BAs). (**E**) Z-score heatmap of serum BA species (primary vs. secondary BAs as indicated). (**F**) Absolute concentrations of representative serum BA species showing overlapping differential patterns as defined in (**D**). (**G**) Hepatic mRNA expression (detected by qPCR) of key enzymes in the classical and non-classical BA synthesis pathways. (**H**,**I**) Western blot (WB) bands (**H**) and quantitative analysis (**I**) of key enzymes in the classical hepatic BA synthesis pathway. (**J**,**K**) WB bands (**J**) and quantitative analysis (**K**) of key enzymes in the non-classical hepatic BA synthesis pathway. Data are presented as mean ± SEM (*n* = 6 mice/group for A–F, *n* = 8 mice/group for G, *n* = 3 mice/group for (**H**–**K**)). Statistical significance was determined by two-way ANOVA with Dunnett’s test (**A**,**D**), unpaired two-tailed Student’s *t*-test (**C**,**F**,**G**), one-way ANOVA with Dunnett’s test (**I**,**K**). * *p* < 0.05, ** *p* < 0.01, *** *p* < 0.001 vs. HFD group.

To evaluate the impact of LGZG on BA metabolism in HFD-induced MASLD mice, we performed targeted BA profiling in both serum and liver, detecting a total of 38 and 39 individual bile acid species, respectively. Compared with chow-fed controls, HFD feeding significantly reduced the total BA pool in the serum (*p* < 0.05), with a similar decreasing trend observed in the liver (Figure 4A,B). Following LGZG treatment, although the total BA pool size remained relatively stable and did not reach statistical significance compared to the HFD group, the intervention significantly remodeled the overall BA compositional profile, shifting it toward a state resembling the control group (Figure 4A,B).

In the liver, the Z-score heatmap showed that HFD broadly suppressed multiple BA species, whereas LGZG largely reversed this trend (Figure 4C). To identify potential metabolic biomarkers, we applied screening criteria of *p* < 0.05 and variable importance in projection (VIP) > 1. Among the 18 BAs significantly altered by HFD, 11 species were significantly restored by LGZG intervention. As shown in Figure 4D, we specifically identified overlapping differential BAs (those significantly downregulated by HFD mice and significantly upregulated after LGZG intervention). In the liver, non-12-OH BAs showed a higher degree of restoration compared to 12OH BAs (8 vs. 3 species, respectively), including 7-DHCA, 12-DHCA, 6,7-DiketoLCA, 7-KetoLCA, 6-ketoLCA, 12-ketoLCA, HCA, and THCA (Figure 4D).

Consistent with hepatic findings, HFD feeding also remodeled the circulating BA profile. The heatmap indicated a clear shift away from the HFD pattern (Figure 4E). Targeted quantification showed that among the 15 BAs significantly downregulated by HFD, 6 species were significantly restored by LGZG. These were predominantly non-12-OH BAs (5 non-12-OH vs. 1 12-OH species), including HCA, 12-DHCA, 6-KetoLCA, 7-KetoLCA, and 7,12-DiketoLCA (Figure 4F).

To elucidate the molecular mechanism underlying these alterations in the BA profile, we analyzed key enzymes involved in hepatic BA synthesis. Both qPCR and WB analyses demonstrated that LGZG did not significantly alter the expression of classical pathway enzymes (CYP7A1 and CYP8B1), which are responsible for 12-OH BA production (*p* > 0.05 vs. HFD) (Figure 4G–I). In contrast, the observed shift in the BA profile was primarily driven by the alternative pathway. LGZG significantly upregulated the expression of alternative pathway enzymes CYP27A1 and CYP7B1, which are critical for generating non-12-OH BAs such as chenodeoxycholic acid (CDCA) and its mouse-specific derivatives, muricholic acids (MCAs) (showing 2.13-fold and 5.08-fold protein increase in CYP27A1 and CYP7B1 expression, respectively, *p* < 0.01 vs. HFD) (Figure 4G,J,K). The restoration of certain 12-OH BAs (e.g., UCA and NorCA) without classical enzyme upregulation may be attributed to LGZG-induced changes in microbial epimerization or intestinal reabsorption rather than de novo hepatic synthesis.

Collectively, these results demonstrate that LGZG promotes non-12OH BA accumulation through selective activation of the alternative hepatic BA synthesis pathway, leading to the consistent enrichment of non-12OH BAs in both hepatic and systemic compartments. Given that several non-12OH BA species are known to exert distinct metabolic effects through G-protein coupled receptor (GPCR) signaling, we next explored their impact on BA receptor expression across key metabolic tissues.

### 2.5. LGZG Promotes Systemic TGR5-GLP-1 Signaling and Modulates the Ileal-Hepatic FXR-FGF15 Axis

**Figure 5:**
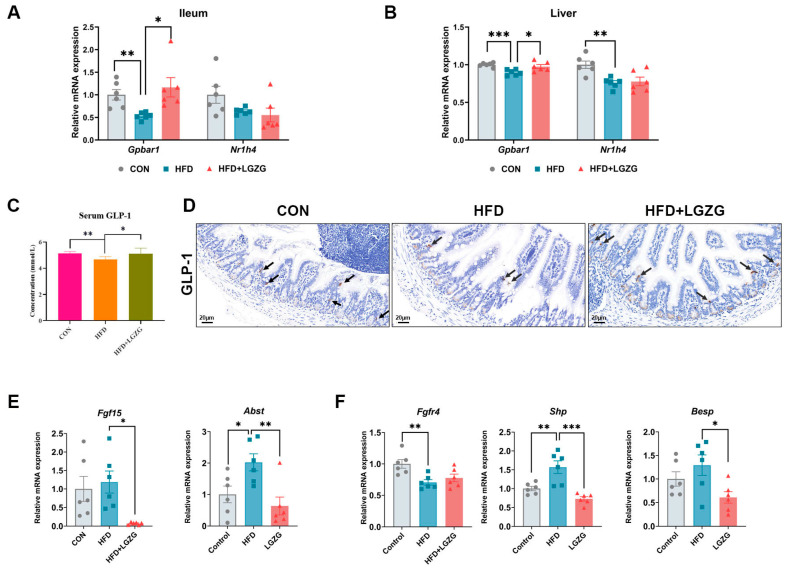
LGZG promotes systemic TGR5 activation with liver-restricted FXR restoration. (**A**) Relative mRNA expression levels of FXR and G-protein coupled bile acid receptor 1 (*TGR5*) in the ileum. (**B**) Relative mRNA expression levels of *FXR* and *TGR5* in the liver. (**C**) Serum GLP-1 levels (*n* = 3). (**D**) Representative immunohistochemical staining of GLP-1 in ileal sections (indicated by black arrows, scale bar = 20 μm, magnification, 400×). (**E**) Relative mRNA expression of intestinal FXR target genes, including *Fgf15* and *Asbt*, in the ileum. (**F**) Relative mRNA expression of hepatic FXR target genes (*Shp*, *Bsep*) and FXR receptor *Fgfr4* in the liver. Data are presented as mean ± SEM (*n* = 6; *n* = 3 for GLP-1 immunohistochemical staining). Statistical significance was determined by two-tailed Student’s *t*-test (**A**–**C**) one-way ANOVA with Dunnett’s test (**E**,**F**). * *p* < 0.05, ** *p* < 0.01, *** *p* < 0.001 vs. HFD group.

Consistent with the observed enrichment of non-12OH BAs (specifically HCAs and LCA derives), we next investigated the expression of key BA receptors, TGR5 (*Gpbar1*) and FXR (*Nr1h4*), across key metabolic tissues. In the ileum, HFD feeding significantly suppressed TGR5 expression (*p* < 0.01), which was markedly restored by LGZG intervention (*p* < 0.05 vs. HFD; Figure 5A). In contrast, ileal *Fxr* mRNA levels remained comparable among all groups (*p* > 0.05; Figure 5A). Concomitantly, LGZG treatment significantly promoted intestinal GLP-1 secretion and increased serum GLP-1 levels (*p* < 0.05; Figure 5C,D), a well-established downstream effect of TGR5 activation that likely underpins in the improved glucose homeostasis observed in Section 2.1. Regarding intestinal FXR activity. Interestingly, despite stable *Fxr* mRNA levels, LGZG intervention significantly downregulated the expression of its downstream target genes—including fibroblast growth factor 15 (*Fgf15*) and apical sodium-dependent bile acid transporter (*Asbt*) (*p* < 0.01 vs. HFD; Figure 5E). This indicated a functional inhibition of the intestinal FXR signaling pathway, possibly mediated by the antagonistic properties of enriched non-12-OH BAs like HCAs in the intestinal lumen.

In the liver, HFD feeding significantly inhibited the expression of both *Tgr5* and *Fxr* (*p* < 0.001 and *p* < 0.01 vs. CON, respectively). LGZG significantly upregulated hepatic *Tgr5* expression (*p* < 0.05 vs. HFD; Figure 5B) but did not significantly alter hepatic *Fxr* mRNA levels. However, functional validation revealed a robust modulation of the hepatic FXR-SHP axis. LGZG intervention markedly suppressed the expression of the FXR corepressor, small heterodimer partner (*Shp*) (*p* < 0.001 vs. HFD), and the bile salt export pump (*Bsep*) (*p* < 0.05 vs. HFD; Figure 5F). Additionally, the expression of Fgfr4, the hepatic receptor for FGF15, remained suppressed in the LGZG group (Figure 5F), consistent with the suppressed ileal Fgf15 expression.

Together, these results support a tissue-specific reprogramming of BA receptor signaling by LGZG, characterized by: (1) systemic TGR5-GLP-1 activation; and (2) a functional shift in the FXR-FGF15/SHP axis that favors reduced bile acid reabsorption and altered feedback regulation. This coordinated receptor modulation, driven by the remodeled BA pool, appears to be a central mechanism underlying the therapeutic effects of LGZG in MASLD.

### 2.6. LGZG Promotes Inguinal White Adipose Tissue Browning via the TGR5/cAMP-PKA-CREB Signaling Axis

Building on the systemic TGR5 activation confirmed in Section 2.5, we investigated whether LGZG exerts metabolic benefits by modulating adipose tissue, a pivotal organ for energy homeostasis. We first quantified the mass of three adipose depots. HFD feeding increased the mass of epididymal white adipose tissue (eWAT), inguinal white adipose tissue (iWAT), and brown adipose tissue (BAT). Notably, LGZG intervention specifically reduced iWAT mass (*p* < 0.01 vs. HFD), while eWAT and BAT weights remained comparable to the HFD group (Figure S1A), indicating a depot-specific regulatory effect.

To evaluate systemic energy metabolism, mice were monitored using the CLAMS. Compared with HFD controls, LGZG-treated mice showed significantly higher oxygen consumption (VO_2_), carbon dioxide production (VCO_2_), and heat production (Figure S1B–D), suggesting an overall elevation in metabolic rate. Importantly, the increase in VCO_2_ was specifically significant during the dark phase (*p* < 0.05; Figure S1C), the period of maximal physical activity for mice.

**Figure 6:**
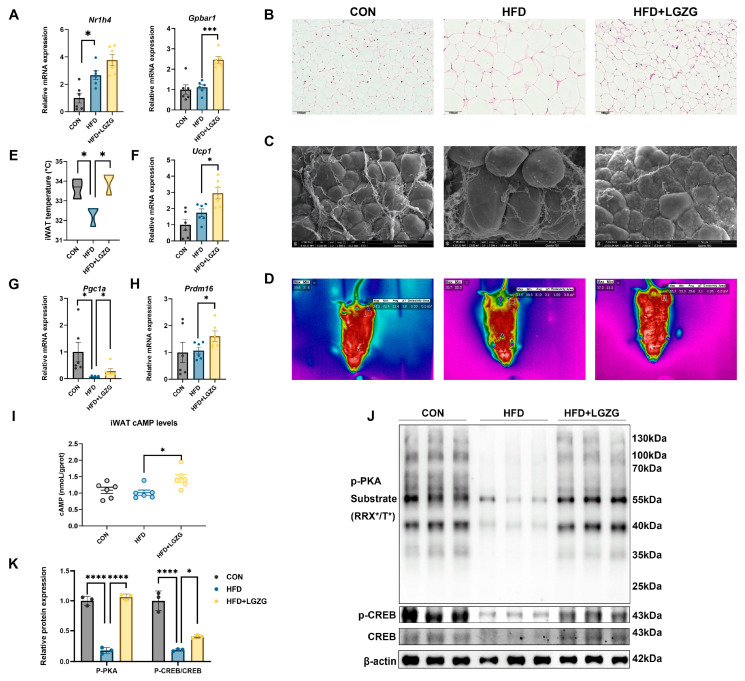
LGZG promotes iWAT browning through activation of TGR5/cAMP-PKA-CREB signaling. (**A**) Relative mRNA expression of FXR and TGR5 in iWAT measured by qPCR. (**B**) Representative H&E-stained iWAT (scale bar = 50 µm). (**C**) Representative SEM images of iWAT showing adipocyte/lipid droplet morphology (scale bar = 10 µm). (**D**) Representative infrared thermal images of the inguinal region (targeting iWAT) (*n* = 3). (**E**) Quantitative analysis of iWAT surface temperature (*n* = 3). (**F**–**H**) Relative mRNA expression levels of key thermogenic genes, including UCP1, PGC1α, and PRDM16 in iWAT measured by qPCR. (**I**) cAMP levels in iWAT measured by enzyme-linked immunosorbent assay (ELISA). (**J**) Representative WB bands showing the protein expression of phosphorylated PKA substrates, cAMP response element binding protein (CREB), and phosphorylated CREB (p-CREB) in iWAT. (**J**,**K**) Quantitative analysis of relative protein expression levels of p-PKA, CREB, and p-CREB in iWAT. Data are presented as mean ± SEM (*n* = 6 for qPCR, WB quantitative analysis and ELISA; *n* = 3 for thermal imaging and temperature measurement). Statistical significance was determined by two-tailed Student’s *t*-test (G and H), one-way ANOVA with Dunnett’s test (**A**,**E**,**F**,**I**,**K**). * *p* < 0.05, *** *p* < 0.001, **** *p* < 0.0001 vs. HFD group.

In iWAT, HFD feeding did not significantly alter *Tgr5* mRNA levels compared with CON mice; however, LGZG intervention robustly upregulated *Tgr5* expression (*p* < 0.001 vs. HFD; Figure 6A). Histological and morphological analyses revealed that LGZG treatment effectively induced a distinct multilocular morphology in iWAT adipocytes (Figure 6B,C) and significantly increased the local surface temperature of iWAT (*p* < 0.05; Figure 6D,E), suggesting enhanced thermogenic activity.

Regarding browning markers, although HFD feeding did not markedly suppress the mRNA expression of *Ucp1* and *Prdm16*, LGZG intervention significantly upregulated their expression (*p* < 0.05 vs. HFD; Figure 6F,H). Moreover, LGZG reversed the HFD-induced downregulation of *Pgc1α* (*p* < 0.05; Figure 6G). Mechanistically, LGZG significantly elevated cAMP levels (*p* < 0.05; Figure 6I) and activated the downstream signaling axis. Western blotting and densitometric quantification (Figure 6J,K) demonstrated that LGZG effectively reversed the HFD-induced suppression of PKA activation and CREB phosphorylation (indicated by increased p-PKA and p-CREB/CREB ratios; *p* < 0.0001 and *p* < 0.001 vs. HFD, respectively).

Collectively, these data demonstrate that LGZG triggers a potent browning program in iWAT via the TGR5/cAMP-PKA-CREB axis, thereby enhancing whole-body energy expenditure and contributing to the alleviation of HFD-induced metabolic disorders.

## 3. Discussion

MASLD represents a complex metabolic disorder characterized by systemic dysregulation and impaired inter-organ communication crosstalk [24]. While emerging therapeutic approaches such as THR-β agonists show promise, single-target agents (e.g., FXR agonists) often encounter limitations, including pruritus and dyslipidemia [4,5,25]. This underscores the critical need for multi-target strategies capable of systemically restoring metabolic homeostasis. As a multi-component herbal formula, our findings demonstrate that the classical Chinese herbal formula LGZG exerted comprehensive therapeutic effects, which appear to be mediated by a coordinated remodeling of the “gut microbiota-bile acid-receptor” axis, accompanied by increased TGR5-preferring non-12-OH bile acids and tissue-specific receptor responses.

In this HFD-induced MASLD mouse model, LGZG administration elicited comprehensive metabolic improvements. Treatment significantly attenuated body weight gain, enhanced glucose tolerance and insulin sensitivity, and ameliorated hypertriglyceridemia (Figure 1B,D–G). Concurrently, LGZG alleviated hepatic injury, as evidenced by reduced serum ALT levels and a decreasing trend in AST levels, lower hepatic lipid accumulation, and improved histopathological scores (Figure 1C and Figure 2A–D). These phenotypic improvements provide the foundation for exploring the underlying mechanisms.

The gut microbiota remodeling induced by LGZG represents a functionally oriented reconstruction rather than a simple reversion to a lean-state profile. Our findings highlight a selective enrichment of key commensals—specifically *Akkermansia*, *Bifidobacterium*, and the *Lachnospiraceae*_NK4A136_group—which exhibited robust negative correlations with hepatic steatosis and injury markers (Figure 3E). The restoration of *Akkermansia* likely fortifies intestinal barrier integrity, thereby limiting endotoxin translocation and downstream hepatic inflammation [26,27]. More critically, the expansion of *Bifidobacterium*, a genus characterized by high bile salt hydrolase (BSH) activity, aligns with the increased demand for primary bile acid deconjugation—a prerequisite for subsequent microbial transformation [28,29]. This expansion of BSH-producing taxa, coupled with the enrichment of the butyrate-producing *Lachnospiraceae*_NK4A136_group, which may optimize the luminal pH to support the catalytic efficiency of bacterial enzymes such as 7α-dehydroxylase and hydroxysteroid dehydrogenases (HSDHs) [30,31,32], thereby facilitating the shift toward a more hydrophobic and signaling-active BA pool. Intriguingly, while *Faecalibacterium* was also elevated by LGZG, it exhibited unexpected positive correlations with NAS and hepatic TG. This suggests that its abundance in this specific model may represent a ‘passenger’ response to altered nutrient availability or dietary lipid substrates—factors common to both HFD and treatment groups—rather than acting as a primary driver of the metabolic recovery observed with LGZG. Similarly, the lack of significant correlation between *Desulfovibrio* or *Lactobacillus* and metabolic phenotypes suggests these taxa might be “passenger” organisms shaped by the altered gut environment, rather than primary “drivers” of LGZG’s therapeutic effects. Collectively, this strategic remodeling favors a microbial consortium that not only preserves gut homeostasis but also provides the specialized enzymatic machinery essential for the subsequent modulation of the bile acid pool.

The most distinctive pharmacological action of LGZG lies in its specific modulation of bile acid synthesis. Unlike broad-spectrum approaches, LGZG selectively activated the alternative pathway by upregulating CYP27A1 and CYP7B1 without affecting CYP7A1 and CYP8B1 (Figure 4H–K). This selective activation is mechanistically profound, as the alternative pathway preferentially generates chenodeoxycholic acid (CDCA) or muricholic acid (MCA), non-12-OH primary bile acids (e.g., CDCA and MCAs) that serve as key biosynthetic precursors for a spectrum of downstream metabolites. By upregulating CYP27A1 and CYP7B1, LGZG expands the hepatic precursor pool for non-12-OH BAs, providing a direct biosynthetic basis for the changes observed in our metabolomic analysis. This hepatic shift is further amplified within the LGZG-modified gut environment. Microbial enzymes, particularly HSDHs from the enriched microbial consortium, may catalyze oxidative modifications generating derivatives such as 6-ketoLCA and 7-ketoLCA (Figure 4A–F). These keto-derivatives not only exhibit substantially reduced cytotoxicity but also demonstrate a biased signaling preference for TGR5 over FXR [33,34,35,36], further amplifying the systemic metabolic benefits of LGZG. It is noteworthy that while LGZG primarily activated the alternative pathway, some 12-OH BAs like UCA were also increased. Given that the expression of the classical pathway enzyme Cyp8b1 remained unchanged, this increase is likely driven by the gut microbiota. Specifically, the LGZG-induced enrichment of 7β-HSDH-producing bacteria (e.g., *Bifidobacterium*) may facilitate the microbial epimerization of CA into UCA. This suggests that LGZG regulates the BA pool through a dual mechanism involving both hepatic enzymatic reprogramming and microbial biotransformation.

Among the enriched non-12-OH BAs, the elevation of the HCA family, including HDCA and their conjugated forms, is particularly noteworthy. HCA, the predominant bile acid in pigs—a species relatively resistant to diet-induced diabetes—has emerged as a potent metabolic regulator. Recent evidence indicates that the HCA family exhibits a unique “dual-target” regulatory profile: they serve as potent systemic TGR5 agonists while acting as natural functional antagonists of intestinal FXR signaling [12]. This dual action perfectly aligns with our observation that LGZG intervention simultaneously upregulated TGR5-GLP-1 signaling and suppressed the ileal FXR-FGF15/Asbt axis. Crucially, the functional inhibition of FXR signaling occurred despite stable Fxr mRNA levels, suggesting that the enrichment of HCA-type antagonists effectively ‘silenced’ the receptor’s transcriptional activity. By enriching the HCA subclass, LGZG facilitates a tissue-specific reprogramming of bile acid signaling that maximizes metabolic benefit while avoiding the potential side effects of systemic FXR over-activation.

LGZG orchestrates sophisticated spatial regulation of bile acid signaling. In peripheral tissues, including the ileum and adipose tissue, selective TGR5 activation drives metabolic benefits. Within ileal L cells, TGR5 engagement stimulates GLP-1 secretion, directly enhancing systemic glucose control [37]. Importantly, our data suggest that LGZG’s effect on adipose tissue is characterized by a potent induction of a compensatory thermogenic program rather than a simple restoration of HFD-induced damage. While HFD feeding did not significantly suppress the basal mRNA levels of browning markers, it effectively induced a state of “functional silencing” of the cAMP-PKA-CREB signaling axis and reduced local iWAT temperature. LGZG-mediated TGR5 activation in iWAT triggered a robust browning response, characterized by reduced adipocyte size, multilocular lipid droplets, and the upregulation of thermogenic genes including *Ucp1*, *Pgc1α*, and *Prdm16*. This enhancement of iWAT thermogenesis contributes to increased systemic energy expenditure, forming a critical component of the multi-targeted therapeutic chain of LGZG (Figure 6).

Hepatic receptor modulation by LGZG follows a distinct, multi-faceted pattern. Our results show that LGZG significantly upregulated hepatic *Tgr5* expression, which is known to exert anti-inflammatory and metabolic benefits [38]. Interestingly, while ileal FXR signaling was functionally suppressed (as evidenced by decreased *Fgf15* and *Asbt*), hepatic FXR-associated downstream targets such as *Shp* and *Bsep* also showed a downward trend (Figure 5). This synchronized suppression in both the intestine and liver likely stems from the enrichment of the HCA family and other non-12-OH BAs, which can act as natural functional antagonists or weak agonists of FXR while robustly activating TGR5. Furthermore, the persistently low expression of hepatic *Fgfr4* in the LGZG group is biologically consistent with the reduced levels of its ileal ligand, FGF15. This tissue-specific modulation strategy—characterized by potent peripheral TGR5 activation coupled with the fine-tuning of hepatic bile acid receptors—exemplifies the integrative pharmacology of LGZG in systemic metabolic recalibration.

The multi-layered mechanism of LGZG challenges conventional single-target approaches. While FXR agonists often induce pruritus and dyslipidemia [4,5,25], LGZG achieves therapeutic efficacy through a “TGR5-biased” signaling profile. Non-12-OH BAs, specifically those enriched by LGZG like the HCA and keto-BA subclasses, exhibit marked receptor selectivity with a significantly higher affinity for TGR5. This inherent signaling specificity enables precise spatial control: TGR5 activation predominates in energy-expending tissues (iWAT) and incretin-secreting cells (L-cells), while the functional antagonism of intestinal FXR signaling prevents the adverse effects associated with systemic over-activation. Moreover, LGZG reconstructs the gut ecosystem by modifying microbial composition and function, facilitating sustained metabolite production that contrasts with the transient pharmacologic effects of synthetic agonists.

Limitations and Future Perspectives: Despite these promising findings, several limitations should be acknowledged. First, while 16S rRNA sequencing identified key microbial shifts, future multi-omics profiling (metagenomics and metatranscriptomics) is essential to identify the bacterial drivers and functional gene clusters (e.g., bsh, 7α-hsdh, or caiABC) governing the biotransformation of non-12-OH BAs. Second, although a strong correlation exists between the gut microbiota and LGZG’s efficacy, experiments involving germ-free (GF) models, fecal microbiota transplantation (FMT), or direct supplementation with specific non-12-OH BAs are required to validate the causal role of the “microbiota–non-12-OH BA” module. Third, considering that non-12-OH BAs are potent endogenous ligands for TGR5, the use of adipose-specific Tgr5 knockout mice will help clarify whether these metabolites promote thermogenesis via direct adipose activation or through broader systemic crosstalk. Finally, a systematic deconstruction of LGZG’s constituents, paired with isotopic tracing of non-12-OH BA flux, will delineate their enterohepatic circulation and tissue-specific biodistribution. These efforts will ultimately decode the pharmacological landscape of LGZG in rebalancing the bile acid pool towards a non-12-OH profile to treat metabolic disorders.

## 4. Materials and Methods

### 4.1. Preparation of LGZG

The preparation of LGZG was performed as previously described. LGZG (batch number: Z201101) were extracted from *Poriacocos* (*Schw.*) Wolf, *Cinnamomum cassia* Presl, *Atractylodes macrocephala* Koidz., and *Glycyrrhiza uralensis* Fisch. in a ratio of 2:1.5:1.5:1. All herbs were purchased from Jiangsu Sanhexing Chinese Medicine Research Co., Ltd. (Lianyungang, China). The crude herbs were soaked in water and subjected to two cycles of reflux extraction with 12 volumes of water, each lasting 1.5 h. The decoction filtrates were combined, and aromatic water collected during extraction was reserved. The filtrate was concentrated to a relative density of 1.07–1.09 (65 ± 5 °C), spray-dried, and ground into fine powder. The yield was 1 g of extract powder per 4.56 g of crude herbs. Quality control of LGZG was performed using fingerprint analysis [21].

### 4.2. Chemicals and Reagents

Experimental Diets: The high-fat diet (HFD; D12492, Research Diets Inc., New Brunswick, NJ, USA) was used to induce the MASLD model, providing an energy density of 5.24 kcal/g. The caloric distribution of the HFD was 60% fat (primarily from lard and soybean oil), 20% protein, and 20% carbohydrate. The control group was fed a standard low-fat diet (CD; D12450B, Research Diets Inc.), containing 10% kcal from fat, 20% protein, and 70% carbohydrate. Rosiglitazone (RSG; purity > 99%, Cat# T6772) was obtained from Topscience Co., Ltd. (Shanghai, China). For histological analysis, Oil Red O (Cat# O0625), hematoxylin solution (Cat# BA-4041), and eosin solution (Cat# BA-4042) were sourced from Sigma-Aldrich (St. Louis, MO, USA) and Baso Diagnostic Inc. (Zhuhai, China), respectively. For immunohistochemistry (IHC) analysis, Tris-EDTA antigen retrieval solution (Cat# AR0023-1) and SABC-POD (Rabbit IgG) Kit (Cat# SA2002) were obtained from Boster Biological Technology Co., Ltd. (Wuhan, China), while the primary antibody against GLP-1 (Cat# GB11335-50) was purchased from Wuhan Servicebio Technology Co., Ltd. (Wuhan, China). ELISA kits for GLP-1 (Cat# CSB-E08118m) and cAMP (Cat# BPE20860) were procured from Cusabio (Wuhan, China) and Lengton (Shanghai, China), respectively. For protein extraction and quantification, radioimmunoprecipitation assay (RIPA) buffer (Cat# P0013B) and bicinchoninic acid (BCA) protein assay kit (Cat# P0012S) were obtained from Beyotime Biotechnology (Shanghai, China). Protease (Cat# 4906837001) and phosphatase (Cat# 4693132001) inhibitor cocktails were purchased from Roche Diagnostics (Basel, Switzerland). For Western blot analysis, polyvinylidene fluoride (PVDF) membranes (Cat# IPFL00010) were obtained from Merck Millipore (Darmstadt, Germany). Primary antibodies against CYP7A1 (Cat# A22897), CYP8B1 (Cat# A25847), CYP27A1 (Cat# A23250), and CYP7B1 (Cat# A17872) were acquired from ABclonal Technology (Wuhan, China). β-actin antibody (Cat# ET1702-67) and horseradish peroxidase (HRP)-conjugated goat anti-rabbit IgG secondary antibody (Cat# HA1001) were purchased from HuaBio (Hangzhou, China). Enhanced chemiluminescence (ECL) substrate (Omni-ECL™ Femto) was obtained from Epizyme, Inc. (Cambridge, MA, USA). For molecular biology experiments, TRIzol reagent (Cat# 15596026CN) was sourced from Thermo Fisher Scientific (Waltham, MA, USA). The 5× Reverse Transcription Master Mix (Cat# AG11706) and SYBR Green Premix Pro Taq HS qPCR Kit (Cat# AG11701) were obtained from Aikeri Biotechnology Co., Ltd. (Changsha, China). All oligonucleotide primers were synthesized by Generay Biotech Co., Ltd. (Shanghai, China), with sequences provided in Supplementary Table S2.

### 4.3. Animals and Experimental Design

Male C57BL/6J mice (7-week-old, weighing 22 ± 2 g) were obtained from GemPharmatech Co., Ltd. (Nanjing, China). Animals were housed under specific pathogen-free (SPF) conditions (temperature 22 ± 1 °C, relative humidity 55 ± 5%, 12 h light/dark cycle) with free access to a standard diet. After a one-week acclimatization period, mice were randomly assigned to four experimental groups (*n* = 8 per group): (1) Control group (CON), (2) HFD-induced MASLD group (HFD), (3) LGZG-treated group (LGZG), and (4) Rosiglitazone-treated group (RSG). To induce MASLD with obesity and hepatic steatosis, the control (CON) group received a standard diet, while the other three groups were fed a HFD for an initial period of 8 weeks. Subsequently, during a 4-week intervention period (from week 9 to week 12), the groups received the following interventions via daily oral gavage: the CON and HFD groups were administered the vehicle; the LGZG group received LGZG at 21.57 g/kg/day (crude drug equivalent, corresponding to 4.73 g/kg/day of the extract powder); and the RSG group received RSG at 10 mg/kg/day as a positive control. The dosage of LGZG was determined based on the human equivalent dose (HED) of 2.37 g/kg/day for a 70 kg adult, converted using the body surface area normalization factor (human: mouse = 1:9.1).

All substances were administered once daily at a volume of 10 mL/kg body weight. Body weight was recorded weekly. At the end of the 12-week experimental period, mice were anesthetized with an intraperitoneal injection of sodium pentobarbital (50 mg/kg). Blood samples were collected via cardiac puncture for serum isolation. The liver, iWAT, and ileum were rapidly excised, weighed, and either fixed in 4% paraformaldehyde, while samples for molecular and biochemical analysis were snap-frozen in liquid nitrogen.

All animal experiments were performed in accordance with the National Institutes of Health (NIH) Guide for the Care and Use of Laboratory Animals and were approved by the Experimental Animal Ethics Committee of Shanghai University of Traditional Chinese Medicine (Approval No.: PZSHUTCM2408260005).

### 4.4. OGTT and ITT

Oral glucose tolerance and insulin tolerance tests were performed as previously described with modifications. For the OGTT, mice were fasted for 12 h and then administered glucose orally (2 g/kg body weight). Blood glucose levels from tail vein blood were measured at 0, 15, 30, 60, 90, and 120 min post-administration using a portable glucometer. For the ITT, mice were fasted for 2 h and received an intraperitoneal injection of insulin (0.75 U/kg body weight), followed by blood glucose measurement at the same time points. The AUC for blood glucose was calculated for both tests.

### 4.5. Detection of Serum Biochemical Indexes

Serum levels of ALT, AST, TG, and TC were measured using an automated biochemical analyzer (TBA-40FR, Toshiba, Tokyo, Japan).

### 4.6. Histological and Immunohistochemical Analysis

#### 4.6.1. H&E Staining and NAFLD Activity Score

Liver and inguinal white adipose tissue (iWAT) samples were fixed in 4% paraformaldehyde, paraffin-embedded, and sectioned at 4 μm. Sections were stained with hematoxylin and eosin (H&E) for histopathological evaluation [39]. Hepatic pathological changes were assessed using the NAFLD Activity Score (NAS) according to the criteria established by Kleiner et al. (2005) [40]. The NAS is defined as the unweighted sum of the scores for steatosis (0–3), lobular inflammation (0–3), and hepatocyte ballooning (0–2), ranging from 0 to 8 [40].

#### 4.6.2. Oil Red O Staining

Frozen liver sections (8 μm) were fixed in 4% paraformaldehyde for 15 min, rinsed with 60% isopropanol, and incubated with Oil Red O working solution for 15 min. After differentiation with 60% isopropanol, nuclei were counterstained with hematoxylin for 1 min. Sections were mounted with aqueous glycerin gelatin. Lipid droplets were observed by light microscopy and quantified using ImageJ (version 1.54h, NIH, Bethesda, MD, USA).

#### 4.6.3. Immunohistochemistry for Intestinal GLP-1

Paraffin-embedded intestinal sections (4 μm) underwent antigen retrieval in Tris-EDTA buffer (pH 9.0), followed by quenching with 3% H_2_O_2_ for 15 min and blocking with 5% normal goat serum at 37 °C for 30 min. Sections were incubated overnight at 4 °C with anti-GLP-1 antibody (1:500), then processed with SABC-POD Kit according to the manufacturer’s instructions. Immunoreactivity was visualized using DAB substrate and counterstained with hematoxylin.

### 4.7. 16S rRNA Gene Sequencing

Gut microbiota profiling was performed via 16S rRNA gene sequencing of intestinal contents from the Control, Model, and LGZG groups. Genomic DNA was extracted using the FastPure Stool DNA Isolation Kit (MJYH, Shanghai, China). The V3-V4 hypervariable region was amplified with primers 338F/806R, and the resulting amplicons were constructed into libraries and sequenced on an Illumina Nextseq2000 platform (Majorbio, Shanghai, China) [41]. Bioinformatic analysis was conducted using the Majorbio Cloud Platform. Briefly, raw sequences were quality-filtered and clustered into operational taxonomic units (OTUs) at 97% similarity. Taxonomic classification was performed against the SILVA database (v138). After rarefying all samples to a depth of 20,000 sequences per sample, alpha and beta diversity indices were calculated. Differentially abundant taxa were identified using Linear Discriminant Analysis Effect Size (LEfSe) with an LDA score > 2 and *p* < 0.05. Functional profiles of the gut microbiota were predicted using PICRUSt2. The 16S rRNA gene sequencing data have been deposited in the NCBI Sequence Read Archive (SRA) under BioProject accession number PRJNA1419534.

### 4.8. Targeted Metabolome Profiling of BAs

Targeted BA profiling of serum and liver samples was performed using the standardized commercial kit (Cat# HY5024R, Shenzhen Bionovogene Co., Ltd., Shenzhen, China) according to the manufacturer’s instructions [42,43,44,45]. This platform is a validated, closed system for the absolute quantification of 66 BA species (detailed in Supplementary Table S1).

For liver sample processing, 10 mg of tissue was homogenized with 25 mg of pre-cooled grinding beads and 20 µL of ultrapure water. The homogenate was then treated similarly to serum samples for protein precipitation. For serum, 50 µL was deproteinized with 400 µL of ice-cold extraction solvent (acetonitrile/methanol, 8:2, *v*/*v*) containing a mixture of stable isotope-labeled internal standards (final concentration of 50 nM). After vortexing (650 rpm, 20 min, 10 °C) and centrifugation, the supernatant was transferred and lyophilized. The dried residue was reconstituted sequentially with 40 µL of acetonitrile/methanol (80:20, *v*/*v*) and 60 µL of deionized water, followed by incubation at −20 °C and a final centrifugation step.

Liquid chromatography-tandem mass spectrometry (UPLC-MS/MS) analysis was performed on a Waters ACQUITY UPLC I-Class system coupled to a Xevo TQ-S mass spectrometer. The MRM transitions, retention times, and collision energies were pre-optimized and integrated into the proprietary analytical platform to ensure high-throughput standardization. A 5 µL aliquot was injected for analysis. Calibration curves (1 to 2000 nM) were prepared using authentic BA standards provided in the kit. Data acquisition and quantitative processing were carried out using MassLynx (v4.1) and iMAP (v1.0) software. This methodology has been previously validated and described in detail [21,46].

### 4.9. RNA Isolation and Real-Time Quantitative PCR

Total RNA was isolated from frozen tissues (liver, iWAT, and ileum) using TRIzol reagent [39]. RNA concentration and purity were determined with a NanoDrop 2000c spectrophotometer (Thermo Fisher Scientific, Wilmington, DE, USA). Equal amounts of qualified RNA were then reverse-transcribed into cDNA using a commercial master mix. Quantitative real-time PCR was subsequently performed on a StepOne Plus Real-Time PCR System with SYBR Green chemistry. The relative expression of target genes was calculated using the comparative threshold cycle (2^−ΔΔCT^) method, with β-actin serving as the internal reference control. The primer sequences are provided in Supplementary Table S2.

### 4.10. Western Blot Analysis

Protein was extracted from liver and iWAT samples. Tissues were homogenized in RIPA lysis buffer containing protease and phosphatase inhibitors, incubated on ice for 30 min, and centrifuged at 12,000× *g* for 15 min at 4 °C. The supernatant was collected, and protein concentration was determined using a BCA assay kit. Equal amounts of protein (20 μg) were separated by 10% SDS-PAGE and transferred to PVDF membranes. After blocking with 5% non-fat milk, the membranes were incubated with specific primary antibodies (Supplementary Table S3) overnight at 4 °C, followed by incubation with HRP-conjugated secondary antibodies (1:20,000). Protein bands were visualized using an ECL substrate and imaged with a Tanon-5200 system [39]. Band intensities were quantified using ImageJ software, and the expression of target proteins was normalized to β-actin.

### 4.11. Infrared Thermal Imaging

Infrared thermal imaging of the ventral inguinal region was performed to assess the surface temperature of iWAT. Mice were lightly anesthetized with isoflurane and placed in a supine position on a thermostatic pad to expose the inguinal area. Thermal images were captured vertically from a fixed height of 30 cm using a FOTRIC 240M thermal imager. Data were analyzed using AnalyzIR software (EasyIR1.0.1), where regions of interest (ROIs) were defined over the bilateral inguinal areas. The maximum or average temperatures within the ROIs were recorded as indicators of iWAT thermogenic activity.

### 4.12. Scanning Electron Microscopy (SEM)

SEM was performed to observe the ultrastructure of iWAT from C57BL/6J mice after 12 weeks of intervention. iWAT samples were dissected and cut into small pieces (approx. 3 × 3 × 1 mm), then fixed in 2.5% glutaraldehyde at 4 °C for over 3 h. After washing with 0.1 M PBS (pH 7.2), tissues were post-fixed with 1% osmium tetroxide (1:1 in 0.2 M PBS) at 4 °C for 2 h, dehydrated through a graded ethanol series (30% to absolute ethanol), critical-point dried, sputter-coated with gold (21 nm), and imaged using a Quanta 250 FEG SEM at 10 kV.

### 4.13. Metabolic Cage Analysis

Whole-body energy metabolism was assessed in C57BL/6J mice after 12 weeks of intervention using a CLAMS (Columbus Instruments, Columbus, OH, USA) over a continuous 2-day period. Mice were individually housed in metabolic chambers with ad libitum access to food and water. Oxygen consumption (VO_2_), carbon dioxide production (VCO_2_), and energy expenditure (EE) were recorded every 15 min. Hourly averages of 24 h cycles were used for statistical analysis. EE was calculated using the Weir equation: EE (kcal/h) = (3.815 + 1.232 × RER) × VO_2_.

### 4.14. Statistical Analysis

Data are presented as mean ± SEM. Statistical analyses were performed using GraphPad Prism (version 10.1.2). Data normality was tested with the Shapiro–Wilk test. For two-group comparisons, unpaired two-tailed Student’s *t*-test was used for normally distributed data; otherwise, the Mann–Whitney U test was applied. For multiple-group comparisons, one-way ANOVA followed by Dunnett’s post hoc test was used for normally distributed data; otherwise, the Kruskal–Wallis test followed by Dunn’s post hoc test was applied. Two-way ANOVA with Dunnett’s multiple comparisons test was used for experiments involving two independent variables. Statistical significance was defined as *p* < 0.05.

Correlations between gut microbiota, bile acid levels, and clinical parameters were assessed by Spearman’s rank correlation analysis with Benjamini–Hochberg FDR correction [47]. Hierarchical clustering and heatmap visualization were performed using GraphPad Prism 10.1.2.

## 5. Conclusions

In summary, our study demonstrates that the classical herbal formula LGZG effectively ameliorates HFD-induced MASLD, as evidenced by significantly reduced hepatic steatosis, lower liver-to-body weight ratios, and normalized serum ALT and AST levels. This therapeutic effect is driven by orchestrating a “gut microbiota–non-12-OH bile acid–TGR5” regulatory axis. We show that LGZG promotes a functional restructuring of the gut microbiota, specifically characterized by the enrichment of *Akkermansia*, *Bifidobacterium*, and *Lachnospiraceae*_NK4A136_group. This microbial shift, coupled with the upregulation of hepatic CYP27A1 and CYP7B1, drives hepatic bile acid synthesis toward the alternative pathway, leading to the selective accumulation of non-12-OH bile acids—most notably the HCA family—which serve as potent TGR5-preferring ligands.

These metabolites exert compartmentalized metabolic benefits across multiple tissues: in the ileum, they stimulate GLP-1 secretion; in the inguinal white adipose tissue (iWAT), they trigger a robust browning program characterized by the upregulation of *Ucp1*, *Pgc1α*, and *Prdm16* via the cAMP-PKA-CREB signaling axis to enhance systemic energy expenditure. Concurrently, this specific bile acid profile facilitates hepatic lipid clearance and suppresses inflammation through balanced FXR/TGR5 modulation, avoiding the adverse effects typically associated with broad-spectrum FXR activation.

Ultimately, our findings provide a mechanistic validation of this traditional formula and elucidate the “gut microbiota–non-12-OH bile acid–TGR5” axis as a promising blueprint for the multi-targeted management of MASLD and other metabolic syndromes. This work bridges the gap between traditional herbal medicine and modern systems biology, offering new insights into next-generation metabolic therapeutics.

**Figure 7:**
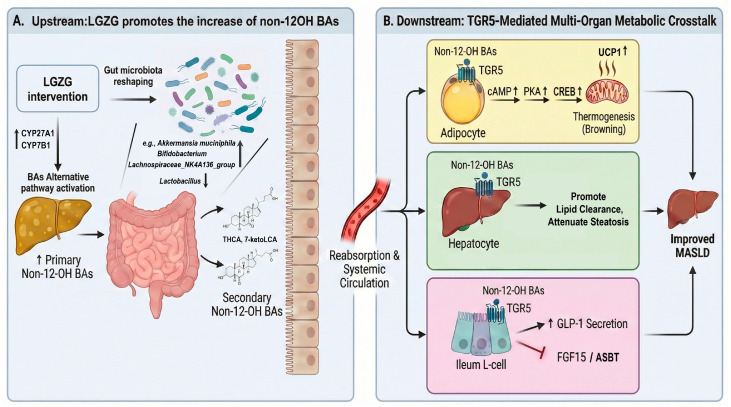
Schematic diagram illustrating the proposed mechanism of LGZG in ameliorating HFD-induced MASLD via the gut microbiota–non-12-OH BA–TGR5 axis. LGZG orchestrates a multi-organ metabolic defense against MASLD through sequential biological modulation. (**A**) In the liver, LGZG shifts bile acid synthesis toward the alternative pathway by upregulating key enzymes (CYP27A1 and CYP7B1), leading to the enrichment of non-12-OH BAs (e.g., HCAs and keto-derivatives). Simultaneously, LGZG reshapes the gut microbiota composition, notably increasing the abundance of commensals such as *Akkermansia* and *Bifidobacterium*, which further optimizes the bile acid profile. (**B**) These TGR5-preferring non-12-OH BAs drive tissue-specific signaling across three key metabolic organs: (1) In adipose tissue (primarily iWAT): LGZG-induced bile acids activate the TGR5-cAMP-PKA-CREB signaling axis, thereby triggering a potent thermogenic program. This is characterized by the induction of multilocular beige adipocytes and the upregulation of thermogenic genes (*Ucp1*, *Pgc1α*, and *Prdm16*), ultimately enhancing systemic energy expenditure; (2) In the liver: LGZG facilitates hepatic TGR5 activation and achieves coordinated modulation of FXR-associated signaling pathways. This dual-receptor engagement promotes lipid clearance and attenuates hepatic steatosis and pathological damage; (3) In the intestine: Non-12-OH BAs selectively activate TGR5 to enhance GLP-1 secretion, improving systemic glucose homeostasis, while concurrently suppressing the intestinal FXR-FGF15 axis (downregulation of *Fgf15* and *Asbt*). Collectively, LGZG ameliorates MASLD not through a single target, but by integrating gut microbial ecology with compartmentalized receptor signaling to restore systemic energy and lipid balance.

Manuscript Preparation: During the preparation of this manuscript, the authors used Gemini 3 pro to create Figure 7, a summary diagram of the mechanism. The authors have reviewed and edited the output and take full responsibility for the content of this publication.

## Data Availability

The original contributions presented in this study are included in the article/supplementary material. Further inquiries can be directed to the corresponding authors.

## Supplementary Materials

The following supporting information can be downloaded at: https://www.mdpi.com/article/10.3390/ph19040523/s1. Figure S1: Whole body energy expenditure and adipose tissue weight changes; Figure S2. UPLC-QTOF-MS analysis of Lingguizhugan decoction. A. Representative ion chromatogram of LGZG in negative mode; B. Representative ion chromatogram of LGZG in positive mode. Table S1. List of 66 Bile Acids and Methodological Parameters for the HY5024R Assay; Table S2: Primer sequences for RT-qPCR; Table S3: Antibodies information; Table S4: Identification results of major components in Lingguizhugan Decoction.

## Abbreviations

The following abbreviations are used in this manuscript:

ALT	Alanine aminotransferase
AST	Aspartate aminotransferase
Asbt	Apical sodium-dependent bile acid transporter
AUC	Area under the curve
BA/BAs	Bile acid(s)
BAT	Brown adipose tissue
BCA	Bicinchoninic acid (protein assay)
Bsep	Bile salt export pump
BSH	Bile salt hydrolase
cAMP	Cyclic adenosine monophosphate
CA	Cholic acid
CDCA	Chenodeoxycholic acid
CLAMS	Comprehensive Lab Animal Monitoring System
ELISA	Enzyme-linked immunosorbent assay
eWAT	Epididymal white adipose tissue
Fgf15	Fibroblast growth factor 15
Fgfr4	Fibroblast growth factor receptor 4
FXR	Farnesoid X receptor
GLP-1	Glucagon-like peptide-1
H&E	Hematoxylin and eosin
HFD	High-fat diet
HSDHs	Hydroxysteroid dehydrogenases
iWAT	Inguinal white adipose tissue
ITT	Insulin tolerance test
LCA	Lithocholic acid
LGZG	Lingguizhugan Decoction
MASLD	Metabolic dysfunction–associated steatotic liver disease
MASH	Metabolic dysfunction–associated steatohepatitis
NAS	NAFLD activity score
Non-12-OH BAs	Non-12α-hydroxylated bile acids
OGTT	Oral glucose tolerance test
PCoA	Principal coordinate analysis
PERMANOVA	Permutational multivariate analysis of variance
PGC1α	peroxisome proliferator-activated receptor gamma coactivator 1-alpha
PRDM16	PR domain containing 16
RSG	Rosiglitazone
RT-qPCR	Reverse transcription quantitative PCR
SEM	Scanning electron microscopy
Shp	Small heterodimer partner
TC	Total cholesterol
TG	Triglycerides
TGR5	G protein-coupled bile acid receptor 1 (GPBAR1)
TRβ	Thyroid hormone receptor beta
UCP1	Uncoupling protein 1
UPLC-MS/MS	Ultra-performance liquid chromatography–tandem mass spectrometry
VCO_2_	Carbon dioxide production
VO_2_	Oxygen consumption

## References

[1] Younossi Z.M., Paik J.M., Stepanova M., Ong J., Alqahtani S., Henry L. (2024). Clinical profiles and mortality rates are similar for metabolic dysfunction-associated steatotic liver disease and non-alcoholic fatty liver disease. J. Hepatol..

[2] Riazi K., Azhari H., Charette J.H., Underwood F.E., King J.A., Afshar E.E., Swain M.G., Congly S.E., Kaplan G.G., Shaheen A.A. (2022). The prevalence and incidence of NAFLD worldwide: A systematic review and meta-analysis. Lancet Gastroenterol. Hepatol..

[3] Eslam M., Newsome P.N., Sarin S.K., Anstee Q.M., Targher G., Romero-Gomez M., Zelber-Sagi S., Wai-Sun Wong V., Dufour J.F., Schattenberg J.M. (2020). A new definition for metabolic dysfunction-associated fatty liver disease: An international expert consensus statement. J. Hepatol..

[4] Harrison S.A., Bedossa P., Guy C.D., Schattenberg J.M., Loomba R., Taub R., Labriola D., Moussa S.E., Neff G.W., Rinella M.E. (2024). A Phase 3, Randomized, Controlled Trial of Resmetirom in NASH with Liver Fibrosis. N. Engl. J. Med..

[5] Younossi Z.M., Ratziu V., Loomba R., Rinella M., Anstee Q.M., Goodman Z., Bedossa P., Geier A., Beckebaum S., Newsome P.N. (2019). Obeticholic acid for the treatment of non-alcoholic steatohepatitis: Interim analysis from a multicentre, randomised, placebo-controlled phase 3 trial. Lancet.

[6] Aron-Wisnewsky J., Vigliotti C., Witjes J., Le P., Holleboom A.G., Verheij J., Nieuwdorp M., Clément K. (2020). Gut microbiota and human NAFLD: Disentangling microbial signatures from metabolic disorders. Nat. Rev. Gastroenterol. Hepatol..

[7] Qin L.N., Yu Y.F., Ma L., Yu R. (2025). Intestinal bacteria-derived extracellular vesicles in metabolic dysfunction-associated steatotic liver disease: From mechanisms to therapeutics. Mol. Cells.

[8] Lee G., Lee J., Suh G.S.B., Oh Y. (2025). Post ingestive systemic nutrient sensing for whole-body homeostasis. Mol. Cells.

[9] Jia W., Li Y., Cheung K.C.P., Zheng X. (2024). Bile acid signaling in the regulation of whole body metabolic and immunological homeostasis. Sci. China Life Sci..

[10] Jia W., Xie G., Jia W. (2018). Bile acid-microbiota crosstalk in gastrointestinal inflammation and carcinogenesis. Nat. Rev. Gastroenterol. Hepatol..

[11] Jia W., Wei M., Rajani C., Zheng X. (2021). Targeting the alternative bile acid synthetic pathway for metabolic diseases. Protein Cell.

[12] Zheng X., Chen T., Jiang R., Zhao A., Wu Q., Kuang J., Sun D., Ren Z., Li M., Zhao M. (2021). Hyocholic acid species improve glucose homeostasis through a distinct TGR5 and FXR signaling mechanism. Cell Metab..

[13] Pathak P., Xie C., Nichols R.G., Ferrell J.M., Boehme S., Krausz K.W., Patterson A.D., Gonzalez F.J., Chiang J.Y.L. (2018). Intestine farnesoid X receptor agonist and the gut microbiota activate G-protein bile acid receptor-1 signaling to improve metabolism. Hepatology.

[14] Sato H., Macchiarulo A., Thomas C., Gioiello A., Une M., Hofmann A.F., Saladin R., Schoonjans K., Pellicciari R., Auwerx J. (2008). Novel potent and selective bile acid derivatives as TGR5 agonists: Biological screening, structure-activity relationships, and molecular modeling studies. J. Med. Chem..

[15] Watanabe M., Houten S.M., Mataki C., Christoffolete M.A., Kim B.W., Sato H., Messaddeq N., Harney J.W., Ezaki O., Kodama T. (2006). Bile acids induce energy expenditure by promoting intracellular thyroid hormone activation. Nature.

[16] Lin H., Zhang Y., Yan H., Wang C., Wen Z., Tong H., Pan X. (2025). Sargassum fusiforme Fucoidan Ameliorates Obesity-Associated Metabolic Dysfunction via a Tauroursodeoxycholic Acid-Mediated TGR5-cAMP-PKA Signaling Pathway. J. Agric. Food Chem..

[17] Velazquez-Villegas L.A., Perino A., Lemos V., Zietak M., Nomura M., Pols T.W.H., Schoonjans K. (2018). TGR5 signalling promotes mitochondrial fission and beige remodelling of white adipose tissue. Nat. Commun..

[18] Liu T., Yang L.L., Zou L., Li D.F., Wen H.Z., Zheng P.Y., Xing L.J., Song H.Y., Tang X.D., Ji G. (2013). Chinese medicine formula lingguizhugan decoction improves Beta-oxidation and metabolism of Fatty Acid in high-fat-diet-induced rat model of Fatty liver disease. Evid. Based Complement. Altern. Med..

[19] Dang Y., Hao S., Zhou W., Zhang L., Ji G. (2019). The traditional Chinese formulae Ling-gui-zhu-gan decoction alleviated non-alcoholic fatty liver disease via inhibiting PPP1R3C mediated molecules. BMC Complement. Altern. Med..

[20] Chen L., Zhang L., Liu S., Hua H., Zhang L., Liu B., Wang R. (2024). Ling-Gui-Zhu-Gan decoction ameliorates nonalcoholic fatty liver disease via modulating the gut microbiota. Microbiol. Spectr..

[21] Zhu M., Wang X., Wang K., Zhao Z., Dang Y., Ji G., Li F., Zhou W. (2023). Lingguizhugan decoction improves non-alcoholic steatohepatitis partially by modulating gut microbiota and correlated metabolites. Front. Cell. Infect. Microbiol..

[22] Tan Y.Y., Yue S.R., Lu A.P., Zhang L., Ji G., Liu B.C., Wang R.R. (2022). The improvement of nonalcoholic steatohepatitis by Poria cocos polysaccharides associated with gut microbiota and NF-kappaB/CCL3/CCR1 axis. Phytomedicine.

[23] Hu P.A., Chen C.H., Guo B.C., Kou Y.R., Lee T.S. (2020). Bromelain Confers Protection against the Non-Alcoholic Fatty Liver Disease in Male C57bl/6 Mice. Nutrients.

[24] Loomba R., Friedman S.L., Shulman G.I. (2021). Mechanisms and disease consequences of nonalcoholic fatty liver disease. Cell.

[25] Adorini L., Trauner M. (2023). FXR agonists in NASH treatment. J. Hepatol..

[26] He K.Y., Lei X.Y., Wu D.H., Zhang L., Li J.Q., Li Q.T., Yin W.T., Zhao Z.L., Liu H., Xiang X.Y. (2023). Akkermansia muciniphila protects the intestine from irradiation-induced injury by secretion of propionic acid. Gut Microbes.

[27] Wu W., Kaicen W., Bian X., Yang L., Ding S., Li Y., Li S., Zhuge A., Li L. (2023). Akkermansia muciniphila alleviates high-fat-diet-related metabolic-associated fatty liver disease by modulating gut microbiota and bile acids. Microb. Biotechnol..

[28] Hidalgo-Cantabrana C., Delgado S., Ruiz L., Ruas-Madiedo P., Sanchez B., Margolles A. (2017). Bifidobacteria and Their Health-Promoting Effects. Microbiol. Spectr..

[29] Xue H., Ma J., Wang Y., Lu M., Wang F., Tang X. (2022). Shen-Ling-Bai-Zhu-San (SL) and SL Derived-Polysaccharide (PL) Ameliorate the Severity of Diarrhea-Induced by High Lactose via Modification of Colonic Fermentation. Front. Pharmacol..

[30] Amedei A., Morbidelli L. (2019). Circulating Metabolites Originating from Gut Microbiota Control Endothelial Cell Function. Molecules.

[31] Fava F., Rizzetto L., Tuohy K.M. (2019). Gut microbiota and health: Connecting actors across the metabolic system. Proc. Nutr. Soc..

[32] Gasaly N., Hermoso M.A., Gotteland M. (2021). Butyrate and the Fine-Tuning of Colonic Homeostasis: Implication for Inflammatory Bowel Diseases. Int. J. Mol. Sci..

[33] Turnbaugh P.J., Ley R.E., Mahowald M.A., Magrini V., Mardis E.R., Gordon J.I. (2006). An obesity-associated gut microbiome with increased capacity for energy harvest. Nature.

[34] Devlin A.S., Fischbach M.A. (2015). A biosynthetic pathway for a prominent class of microbiota-derived bile acids. Nat. Chem. Biol..

[35] Perino A., Schoonjans K. (2015). TGR5 and Immunometabolism: Insights from Physiology and Pharmacology. Trends Pharmacol. Sci..

[36] Collins S.L., Stine J.G., Bisanz J.E., Okafor C.D., Patterson A.D. (2023). Bile acids and the gut microbiota: Metabolic interactions and impacts on disease. Nat. Rev. Microbiol..

[37] Song M., Chan A.T. (2019). Environmental Factors, Gut Microbiota, and Colorectal Cancer Prevention. Clin. Gastroenterol. Hepatol..

[38] Shi Y., Su W., Zhang L., Shi C., Zhou J., Wang P., Wang H., Shi X., Wei S., Wang Q. (2020). TGR5 Regulates Macrophage Inflammation in Nonalcoholic Steatohepatitis by Modulating NLRP3 Inflammasome Activation. Front. Immunol..

[39] Li M., Wang Y.R., Wang X., Xiao X.L., Sun Y.H., Zhang S.A., Dang Y.Q., Wang K., Zhou W.J. (2025). Emodin Enhances Rosiglitazone’s Therapeutic Profile by Dual Modulation of SREBP1-Mediated Adipogenesis and PPARgamma-Driven Thermogenesis. Pharmaceuticals.

[40] Kleiner D.E., Brunt E.M., Van Natta M., Behling C., Contos M.J., Cummings O.W., Ferrell L.D., Liu Y.C., Torbenson M.S., Unalp-Arida A. (2005). Design and validation of a histological scoring system for nonalcoholic fatty liver disease. Hepatology.

[41] Shu X., Li M., Cao Y., Li C., Zhou W., Ji G., Zhang L. (2021). Berberine Alleviates Non-alcoholic Steatohepatitis Through Modulating Gut Microbiota Mediated Intestinal FXR Activation. Front. Pharmacol..

[42] Xie G., Zhong W., Li H., Li Q., Qiu Y., Zheng X., Chen H., Zhao X., Zhang S., Zhou Z. (2013). Alteration of bile acid metabolism in the rat induced by chronic ethanol consumption. FASEB J..

[43] Xie G., Wang Y., Wang X., Zhao A., Chen T., Ni Y., Wong L., Zhang H., Zhang J., Liu C. (2015). Profiling of serum bile acids in a healthy Chinese population using UPLC-MS/MS. J. Proteome Res..

[44] Yin S., Su M., Xie G., Li X., Wei R., Liu C., Lan K., Jia W. (2017). Factors affecting separation and detection of bile acids by liquid chromatography coupled with mass spectrometry in negative mode. Anal. Bioanal. Chem..

[45] Zhu P., Zhang J., Chen Y., Yin S., Su M., Xie G., Brouwer K.L.R., Liu C., Lan K., Jia W. (2018). Analysis of human C24 bile acids metabolome in serum and urine based on enzyme digestion of conjugated bile acids and LC-MS determination of unconjugated bile acids. Anal. Bioanal. Chem..

[46] She J., Tuerhongjiang G., Guo M., Liu J., Hao X., Guo L., Liu N., Xi W., Zheng T., Du B. (2024). Statins aggravate insulin resistance through reduced blood glucagon-like peptide-1 levels in a microbiota-dependent manner. Cell Metab..

[47] Han J.X., Tao Z.H., Wang J.L., Zhang L., Yu C.Y., Kang Z.R., Xie Y., Li J., Lu S., Cui Y. (2023). Microbiota-derived tryptophan catabolites mediate the chemopreventive effects of statins on colorectal cancer. Nat. Microbiol..

