# Supplementary material for: Lingguizhugan Decoction Ameliorates MASLD by Modulating the Gut Microbiota and Enriching Non-12-OH Bile Acids to Activate TGR5-Mediated Thermogenesis
Source: Pharmaceuticals (Basel). 2026 Mar 24;19(4):523. doi: 10.3390/ph19040523 (PMC13118965; doi:10.3390/ph19040523)
Supplement: Supplementary file 1 [file pharmaceuticals-19-00523-s001.zip › pharmaceuticals-4172017-supplementary.pdf]

Lingguizhugan Decoction Ameliorates MASLD by Modulating the Gut Microbiota and Enriching Non - 12 - OH Bile Acids to Activate TGR5 - Mediated Thermogenesis

Yun-Hong Sun [1,4,†], Pei-Lun Ding [1,†], Xue Wang [1,5], Yi-Rong Wang [1], Ming-Zhe Zhu [4], Kai Wang [6], Liang Dai[1,2,4], Yan-Qi Dang [1,2], Guang Ji [1,2], and Meng Li [1,2,3,*], and Wen-Jun Zhou [1,2,*]

1 Institute of Digestive Diseases, Shanghai University of Traditional Chinese Medicine, Shanghai 200032, China;

2 State Key Laboratory of Integration and Innovation of Classical Formula and Modern Chinese Medicine, Shanghai 201203, China;

3 School of Biological Sciences, Nanyang Technological University, Singapore 637551, Singapore;

4 School of Public Health, Shanghai University of Traditional Chinese Medicine, Shanghai 201203, China

5 CAS Engineering Laboratory for Nutrition, Shanghai Institute of Nutrition and Health, University of Chinese Academy of Sciences, Chinese Academy of Sciences, Shanghai 200031, China;

6 Experiment Center for Science and Technology, Shanghai University of Traditional Chinese Medicine, Shanghai 201203, China;

* Correspondence:Wen-Jun Zhou: zhouwenjun@shutcm.edu.cn; Meng-Li: mengli@ntu.edu.sg

† These authors contributed equally to this work.

Supplementary Figure

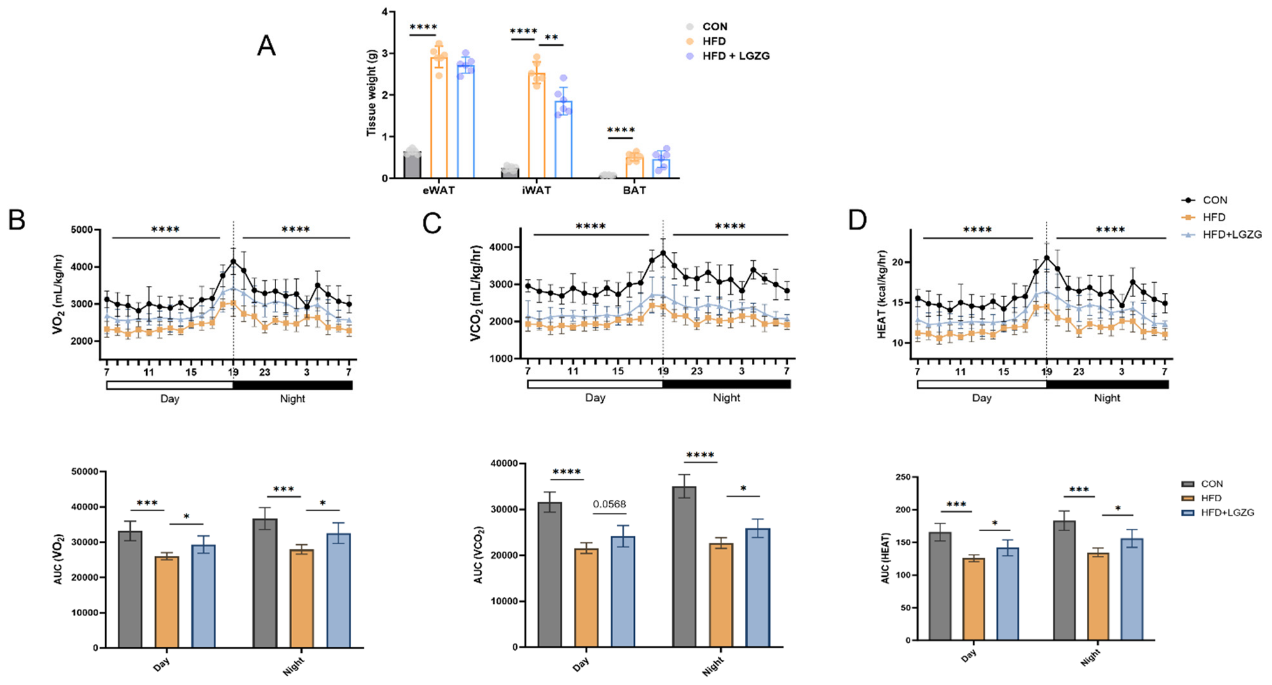

Figure S1. Whole body energy expenditure and adipose tissue weight changes. (A) Tissue weights of epididymal white adipose tissue (eWAT), inguinal white adipose tissue (iWAT), and brown adipose tissue (BAT) after 12 weeks of intervention. (B-D) Systemic energy metabolism parameters monitored by the Comprehensive Lab Animal Monitoring System (CLAMS) over a 24-hour period: (B) oxygen consumption rate (VO_2), (C) carbon dioxide production rate (VCO_2), and (D) heat production. Data are presented as mean ± SEM (n = 6/group). Statistical significance was determined by two-tailed Student's *t*-test. $^{*}p < 0.05$, $^{**}p < 0.01$, $^{***}p < 0.001$, $^{****}p < 0.0001$ vs. HFD group

Supplementary Tables

Supplementary Table S1. List of 66 Bile Acids and Methodological Parameters for the HY5024R Assay

Analyte	Abbreviation	HMDB	LOD (nmol/L)	LOQ (nmol/L)	Linearity range (nmol/L)
7α-Hydroxy-4-Cholesten-3-One	C4	HMDB0001993	1.5	5	5-2000
3-Oxolithocholic Acid	3-oxoLCA/DHLCA	N/A	1.5	5	5-2000
Isoallolithocholic Acid	IsoalloLCA	HMDB0000713	1.5	5	5-2000
Isolithocholic Acid	IsoLCA	HMDB0000717	1.5	5	5-2000
Lithocholic Acid	LCA	HMDB0000761	1.5	5	5-2000
Nordeoxycholic Acid	NorDCA	HMDB0304947	1.5	5	5-2000
6-Ketolithocholic Acid	6-ketoLCA	N/A	1.5	5	5-2000
7-Ketolithocholic Acid	7-ketoLCA	HMDB0000467	1.5	5	5-2000
12-Ketolithocholic Acid	12-ketoLCA	HMDB0000328	1.5	5	5-2000
Apocholic Acid	ApoCA	N/A	1.5	5	5-2000
Murocholic Acid	muroCA	HMDB0000811	1.5	5	5-2000
Isoursodeoxycholic Acid	βUDCA	HMDB0000686	1.5	5	5-2000
Isohyodeoxycholic Acid	βHDCA	HMDB0000664	1.5	5	5-2000
Ursodeoxycholic Acid	UDCA	HMDB0000946	1.5	5	5-2000
Hyodeoxycholic Acid	HDCA	HMDB0000733	1.5	5	5-2000
3β-Deoxycholic Acid	βDCA	HMDB0000438	1.5	5	5-2000
Chenodeoxycholic Acid	CDCA	HMDB0000518	1.5	5	5-2000
Deoxycholic Acid	DCA	HMDB0000626	1.5	5	5-2000
Isodeoxycholic Acid	IsoDCA	HMDB0002536	1.5	5	5-2000
Norcholic Acid	NorCA	HMDB0255727	1.5	5	5-2000
Dehydrocholic Acid	DHCA	N/A	1.5	5	5-2000
6,7-Diketolithocholic Acid	6,7-diketoLCA	N/A	1.5	5	5-2000
7,12-Diketolithocholic Acid	7,12-diketoLCA	N/A	1.5	5	5-2000
12-Dehydrocholic Acid	12-DHCA	N/A	1.5	5	5-2000

Analyte	Abbreviation	HMDB	LOD (nmol/L)	LOQ (nmol/L)	Linearity range (nmol/L)
7-Dehydrocholic Acid	7-DHCA	N/A	1.5	5	5-2000
3-Oxocholic Acid	3-DHCA	HMDB0000502	1.5	5	5-2000
Ursocholic Acid	UCA	HMDB0000917	1.5	5	5-2000
3β-Cholic Acid	βCA	HMDB0000419	1.5	5	5-2000
α-Muricholic Acid	αMCA	HMDB0000506	1.5	5	5-2000
β-Muricholic Acid	βMCA	HMDB0000415	1.5	5	5-2000
ω-Muricholic Acid	ωMCA	HMDB0000364	1.5	5	5-2000
Hyocholic Acid	HCA	HMDB0000760	1.5	5	5-2000
Allocholic Acid	ACA	HMDB0000505	1.5	5	5-2000
Cholic Acid	CA	HMDB0000619	1.5	5	5-2000
Glycodehydrocholic Acid	GDHCA	N/A	1.5	5	5-2000
Glycolithocholic Acid	GLCA	HMDB0000698	1.5	5	5-2000
Glycoursodeoxycholic Acid	GUDCA	HMDB0000708	1.5	5	5-2000
Glycohyodeoxycholic Acid	GHDCA	HMDB0304944	1.5	5	5-2000
Glycochenodeoxycholic Acid	GCDCA	HMDB0000637	1.5	5	5-2000
Glycodeoxycholic Acid	GDCA	HMDB0000631	1.5	5	5-2000
Glycohyocholic Acid	GHCA	HMDB0242179	1.5	5	5-2000
Glycocholic Acid	GCA	HMDB0000138	1.5	5	5-2000
Taurodehydrocholic Acid	TDHCA	HMDB0258743	1.5	5	5-2000
Taurolithocholic Acid	TLCA	HMDB0000722	1.5	5	5-2000
Tauroursodeoxycholic Acid	TUDCA	HMDB0000874	1.5	5	5-2000
Taurohyodeoxycholic Acid	THDCA	N/A	1.5	5	5-2000
Taurodeoxycholic Acid	TDCA	HMDB0000896	1.5	5	5-2000
Taurochenodeoxycholic Acid	TCDCA	HMDB0000951	1.5	5	5-2000
Tauro-ω-Muricholic Acid	TωMCA	N/A	1.5	5	5-2000
Tauro-α-Muricholic Acid	TαMCA	HMDB0258742	1.5	5	5-2000
Tauro-β-Muricholic Acid	TβMCA	HMDB0000932	1.5	5	5-2000
Taurohyocholic Acid	THCA	HMDB0011637	1.5	5	5-2000
Taurocholic Acid	TCA	HMDB0000036	1.5	5	5-2000
Lithocholic Acid 3 Sulfate	LCA-3S	HMDB0000907	1.5	5	5-2000

Analyte	Abbreviation	HMDB	LOD (nmol/L)	LOQ (nmol/L)	Linearity range (nmol/L)
7-Sulfo-Ursodeoxycholic Acid	UDCA-7S	N/A	1.5	5	5-2000
3-Sulfodeoxycholic Acid	DCA-3S	HMDB0002504	1.5	5	5-2000
3-Sulfo-Glycodeoxycholic Acid	GDCA-3S	N/A	1.5	5	5-2000
Glycochenodeoxycholic Acid 3-Sulfate	GCDCA-3S	HMDB0002497	1.5	5	5-2000
Glycolithocholic Acid 3 Sulfate	GLCA-3S	HMDB0002639	1.5	5	5-2000
Taurolithocholic Acid 3 Sulfate	TLCA-3S	HMDB0002580	1.5	5	5-2000
Deoxycholic Acid 3-Glucuronide	DCA-3Glu	HMDB0002596	1.5	5	5-2000
Chenodeoxycholic Acid-3-β-D-Glucuronide	CDCA-3Glu	HMDB0002430	1.5	5	5-2000
Chenodeoxycholic Acid 24-Acyl-β-D-Glucuronide	CDCA-24Glu	N/A	1.5	5	5-2000
Glycodeoxycholic Acid-3-O-β-Glucuronide	GDCA-3Glu	N/A	1.5	5	5-2000
Glycochenodeoxycholic Acid-3-O-β-Glucuronide	GCDCA-3Glu	N/A	1.5	5	5-2000
β-Chenodeoxycholic Acid	βCDCA	N/A	1.5	5	5-2000

Supplementary Table S2. Primer sequences for RT-qPCR

Primers	Forward	Reverse
Actb (β-Actin)	GAGACCTTCAACACCCCAGC	ATGTCACGCACGATTTCCC
Cyp7a1	GGGATTGCTGTGGTAGTGAGC	GGTATGGAATCAACCCGTTGTC
Cyp8b1	GGTACGCTTCCTCTATCGCC	GAGGGATGGCGTCTTATGGG
Cyp27a1	CCAGGCACAGGAGAGTACG	GGGCAAGTGCAGCACATAG
Cyp7b1	TGAAATAGGAGCACATCATCTTGG	AATACATTGCCCAGAACATAGCTG
Gpbar1 (Tgr5)	TGCTTCTTCCTAAGCCTACTACT	CTGATGGTTCCGGCTCCATAG
Nr1h4 (Fxr)	GGCAGAATCTGGATTTGGAATCG	GCCCAGGTTGGAATAGTAAGACG
Nr0b2 (Shp)	TCTGCAGGTCGTCCGACTATTC	AGGCAGTGGCTGTGAGATGC
Abcb11 (Besp)	ACATTGGCTCCATAGAGCAAGC	TGCCTGGACAAAGCCAAATCCT
Fgf15	GACTGCGAGGAGGACCAAAA	CAGCCCGTATATCTTGCCGT
Fgfr4	TCCATGACCGTCGTACACAAT	ATTTGACAGTATTCCCGGCAG
Slc10a2 (Asbt)	TCCTCTGTCTGTCCCCCAAA	GGAAGCCCACGAAGATACCC
Ppara	TATTCGGCTGAAGCTGGTGTAC	CTGGCATTTGTTCCGGTTCT
Srebf1 (Srebp1c)	GGAGATGCTATCTCATGGCA	GAAACGTGTCAAGTGCAGG
Srebf2 (Srebp2)	GCAGCAACGGGACCATTCT	CCCCATGACTAAGTCCTTCAACT
Hmgcr	ATCAGTGACACCCCTGCTTG	ACACAGCACGGAAAGAACCA
Prdm16	CACTCCCTCTACCCCTTTACG	CGGGTTTGGCCTCTTTTG
Ucp1	CGGCTTAATGACTGGAGGTG	GCATTGTAGGTCCCCGTGTAG
Ppargc1a (Pgc1a)	TGGCACGCAGCCCTATTC	GAGGATCTACTGCCTGGGGAC
Adrb2	CTCAGGAACGGGACGAAGC	CACGCCAAGGAGATTATGAAGTAG
Adrb3	CGAAACTGGTTGCGAACTGTG	GGTAACCAGCGTGCCGTAAC

Supplementary Table S3. Antibodies information. Abbreviations: WB, western blot

Antibody	Catalog number	Company	Use
**β-ACTIN**	ET1702-67	HUABIO	WB
**CYP7A1**	A22897	ABclonal	WB
**CYP8B1**	A25847	ABclonal	WB
**CYP27A1**	A23250	ABclonal	WB
**CYP7B1**	A17872	ABclonal	WB
**P-PKA**	9624	CST	WB
**P-CREB**	9198S	CST	WB
**CREB**	9197S	CST	WB

A

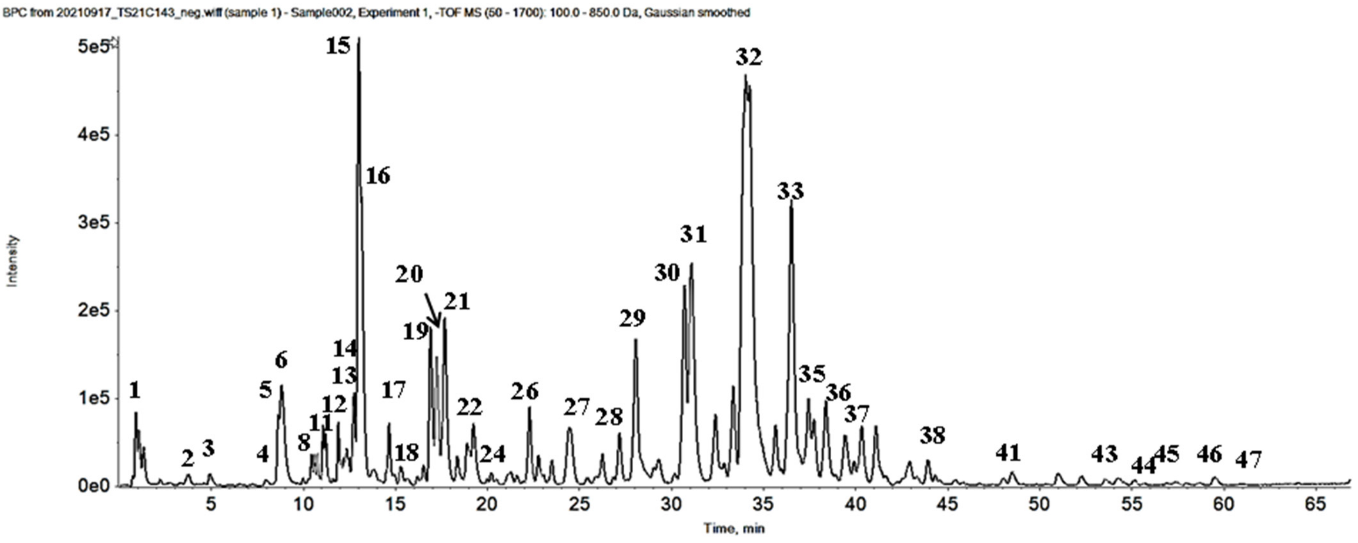

B

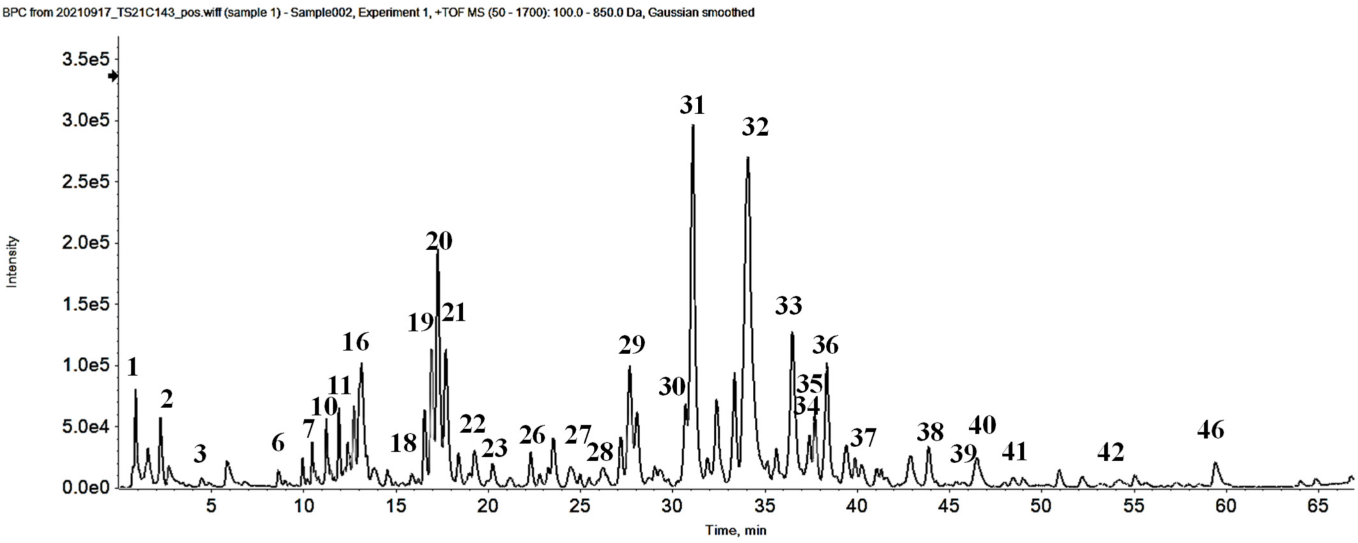

Figure S2. UPLC-QTOF-MS analysis of Lingguizhugan decoction. A. Representative ion chromatogram of LGZG in negative mode; B. Representative ion chromatogram of LGZG in positive mode.

Supplementary Table S4. Identification results of major components in Lingguizhugan Decoction

No.	RT (min)	Adduct ion	Measured m/z	Calculated m/z	ppm	Molecular formula	Molecular weight	Identification	MS/MS data
1	1.072	$[M-H]^-$	191.0209	191.0197	6.3	$C_6H_8O_7$	192.12	Citric acid	111.0089;87.0087;85.0295;67.0187
2	2.342	$[M-H]^-$	169.0156	169.0142	8.3	$C_7H_6O_5$	170.12	Gallic acid	124.0160;95.0120;79.0186;67.0184;51.0237
3	4.957	$[M-H]^-$	153.0202	153.0193	5.9	$C_7H_6O_4$	154.12	Protocatechuic acid	108.0211
4	7.948	$[M-H]^-$	137.0255	137.0244	8.0	$C_7H_6O_3$	138.12	Protocatechualdehyde	108.0211;92.0265
5	8.8	$[M-H]^-$	209.0459	209.0455	1.9	$C_{10}H_{10}O_5$	210.18	5-Hydroxyferulic acid	165.0562;121.0656;119.0494;93.0342
6	9.05	$[M+NH4]^+$	466.2639	466.2647	-1.7	$C_{21}H_{36}O_{10}$	448.5	Atractyloside A	466.1934;269.1736;251.1625;233.0530
7	10.43	$[M-H]^-$	431.1226	431.1195	7.2	$C_{18}H_{24}O_{12}$	432.38	Licoagroside B	431.1234;287.0825;161.0467;125.0255
8	10.6	$[M-H]^-$	417.1208	417.1191	4.1	$C_{21}H_{22}O_9$	418.39	Neoliquiritin	417.1188;255.0663;135.0090;119.0503
9	10.76	$[M-H]^-$	711.2159	711.2142	2.4	$C_{32}H_{40}O_{18}$	712.65	Glucoliquiritin apioside	711.2152;549.1630;255.0678;135.0094
10	11.2	$[M-H]^-$	593.1546	593.1512	5.7	$C_{27}H_{30}O_{15}$	594.52	Vicenin-2	593.1496;473.1102;383.0755;353.0659
11	11.89	$[M-H]^-$	563.1448	563.1406	7.5	$C_{26}H_{28}O_{14}$	564.49	Schaftoside	563.1423;473.1118;443.1006;383.0782;353.0676
12	12.06	$[M-H]^-$	581.2271	581.224	5.3	$C_{28}H_{38}O_{13}$	582.59	(-)-Lyoniresinol 9'-O-glucoside	581.2266;419.1707;404.1514;373.1280
13	12.2	$[M-H]^-$	581.2264	581.224	4.1	$C_{28}H_{38}O_{13}$	582.59	(+)-Lyoniresinol-3-α-O-β-D-glucopyranoside	581.2268;419.1732;404.1497;373.1322;255.0672
14	12.74	$[M-H]^-$	549.1654	549.1614	7.3	$C_{26}H_{30}O_{13}$	550.51	Liguiritigenin-7-O -apiosyl-4′-O-D-glucoside	549.1639;429.1068;255.0673;135.0097
15	13.01	$[M-H]^-$	549.1643	549.1614	5.3	$C_{26}H_{30}O_{13}$	550.51	Liquiritin apioside	549.1626;429.1202;255.0661;135.0087
16	13.14	$[M-H]^-$	417.1213	417.1191	5.3	$C_{21}H_{22}O_9$	418.39	Liquiritin	417.1184;255.0663;135.0087;119.0504

17	14.64	$[M-H]^-$	579.1413	579.1355	10.0	$C_{26}H_{28}O_{15}$	580.49	Quercetin 3-[rhamnosyl-(1→2)-α-L-arabinopyranoside]	579.1373;533.2655;429.0885;301.0339;300.0281
18	15.586	$[M+H]^+$	147.0448	147.0441	4.8	$C_9H_6O_2$	146.14	Coumarin	147.0440;91.0541;65.0383
19	16.92	$[M-H]^-$	549.1644	549.1614	5.5	$C_{26}H_{30}O_{13}$	550.51	Isoliquiritin apioside	549.1648;417.1232;255.0668;135.0091
20	17.25	$[M+FA-H]^-$	475.1276	475.1246	6.3	$C_{22}H_{22}O_9$	430.4	Ononin	475.1295;267.0681;252.0444
21	17.69	$[M-H]^-$	417.1217	417.1191	6.2	$C_{21}H_{22}O_9$	418.39	Isoliquiritin	417.1189;255.0662;135.0085;119.0497
22	19.23	$[M-H]^-$	255.0673	255.0663	3.9	$C_{15}H_{12}O_4$	256.25	Liquiritigenin	255.0665;135.0100;119.0510;91.0199
23	19.95	$[M+H]^+$	149.059	149.0597	-4.7	$C_9H_8O_2$	148.16	Cinnamic acid	131.0473;103.0526;77.0377
24	20.21	$[M-H]^-$	837.3975	837.3914	7.3	$C_{42}H_{62}O_{17}$	838.93	Macedonoside A isomer	837.3943;351.0570;193.0355
25	21.71	$[M+H]^+$	133.0639	133.0648	-6.8	C_9H_8O	132.16	Cinnamaldehyde	133.0647;115.0546;103.0541;77.0374
26	22.27	$[M-H]^-$	823.4177	823.4122	6.7	$C_{42}H_{64}O_{16}$	824.95	Licorice saponin J2	823.4156
27	24.43	$[M-H]^-$	821.4005	821.3965	4.9	$C_{42}H_{62}O_{16}$	822.93	Licorice saponin K2	821.4056;645.3834;351.0601;289.0569
28	26.21	$[M+H]^+$	985.4691	985.4639	5.3	$C_{48}H_{72}O_{21}$	985.07	Licorice saponin A3	985.4682;809.4340;615.3888;453.3358
29	27.67	$[M-H]^-$	879.4096	879.402	8.6	$C_{44}H_{64}O_{18}$	880.97	22β -Acetoxyglycyrrhizin	879.4011;351.0571;193.0352
30	30.69	$[M-H]^-$	819.3864	819.3809	6.7	$C_{42}H_{60}O_{16}$	820.92	Licorice saponin E2	819.3774;643.3510;351.0563;193.0349
31	31.1	$[M-H]^-$	837.3956	837.3914	5.0	$C_{42}H_{62}O_{17}$	838.93	Yunganoside K2	837.3876;775.3872;351.0563;289.0573
32	34.26	$[M-H]^-$	821.4025	821.3965	7.3	$C_{42}H_{62}O_{16}$	822.93	Glycyrrhizic acid	821.4003;759.3957;645.3681;351.0589
33	36.48	$[M-H]^-$	821.4027	821.3965	7.5	$C_{42}H_{62}O_{16}$	822.93	Licorice saponin H2	821.3985;645.3647;351.0572;289.0581
34	37.29	$[M+H]^+$	249.1464	249.1485	-8.4	$C_{15}H_{20}O_3$	248.32	Atractylenolide III	231.1391;189.0918;163.0757;135.0425

35	37.45	[M-H]-	821.4039	821.3965	9.0	$C_{42}H_{62}O_{16}$	822.93	Uralsaponin B	821.4021;645.3714;351.0600;289.0594
36	38.42	[M-H]-	823.4194	823.4122	8.7	$C_{42}H_{64}O_{16}$	824.95	Licorice saponin J2	823.4174;777.4146;351.0573
37	40.34	[M-H]-	805.4074	805.4016	7.2	$C_{42}H_{62}O_{15}$	806.93	Licorice saponin C2	805.4078;743.4001;351.0576
38	43.93	[M-H]-	645.3705	645.3644	9.5	$C_{36}H_{54}O_{10}$	646.81	Glycyrrhetinic acid 3-O-mono-β-D-glucuronide	645.3715;469.3374
39	45.75	[M+H]+	233.1547	233.1536	4.7	$C_{15}H_{20}O_2$	232.32	Atractylenolide Ⅱ isomer	233.1549;215.1445;187.1481;177.0906;159.0786
40	46.5	[M+H]+	233.1541	233.1536	2.1	$C_{15}H_{20}O_2$	232.32	Atractylenolide Ⅱ	/
41	48.45	[M-H]-	351.0891	351.0874	4.8	$C_{20}H_{16}O_6$	352.34	Licoisoflavone B	351.0914;307.1006;283.1007;265.0873
42	53.15	[M+H]+	231.1369	231.138	-4.8	$C_{15}H_{18}O_2$	230.3	Atractylenolide Ⅰ	231.1381;213.1291;203.1085;185.1321;157.1003
43	54.07	[M-H]-	483.3164	483.3111	11.0	$C_{30}H_{44}O_5$	484.32	Poricoic acid B	483.3106;409.2732;211.1503
44	57.491	[M-H]-	497.3335	497.3267	13.7	$C_{31}H_{46}O_5$	498.33	Poricoic acid A	497.3235;423.2881;213.1263
45	57.845	[M-H]-	485.3319	485.3272	9.7	$C_{30}H_{46}O_5$	486.33	Poricoic acid G	441.3352;355.2619;303.2298
46	59.46	[M-H]-	469.3368	469.3323	9.6	$C_{30}H_{46}O_4$	470.68	16α-Hydroxydehydrotrametenolic acid	469.3361;425.3457;409.3131;355.2693
47	60.863	[M-H]-	481.3338	481.3323	3.1	$C_{31}H_{46}O_4$	482.34	Polyporenic acid C	481.3293;85.0656
